# TOX High-Mobility Group Box Family Member 4 promotes DNA double-strand break repair *via* nonhomologous end joining

**DOI:** 10.1016/j.jbc.2025.110174

**Published:** 2025-05-04

**Authors:** Feifei Wang, Wenli Gui, Mengtao Rong, Liang Zhang, Jiajing Wu, Juan Li, Renqing Wang, Odjo G. Gouttia, Ling Wang, Xingyuan Yang, Aimin Peng

**Affiliations:** 1 Institute of Health Sciences and Technology, Institutes of Physical Sciences and Information Technology, Anhui University, Hefei, Anhui, China; 2 Department of Orthopedics, Affiliated Hospital of Jiujiang University, Jiujiang, Jiangxi, PR China; 3 Department of Pathology, The First Affiliated Hospital of Anhui Medical University, Hefei, Anhui, China; 4 Department of Biomedical Sciences, Adams School of Dentistry, University of North Carolina at Chapel Hill, Chapel Hill, North Carolina, USA; 5 Lineberger Comprehensive Cancer Center, University of North Carolina at Chapel Hill, Chapel Hill, North Carolina, USA

**Keywords:** TOX4, NHEJ, DNA-PKcs, DNA repair, PNUTS

## Abstract

Nonhomologous end joining (NHEJ) is a pivotal mechanism in the repair of DNA double-strand breaks. Central to NHEJ is the DNA-dependent protein kinase (DNA-PK) complex, comprising the KU heterodimer and the catalytic subunit, DNA-PKcs. In this study, we characterize Thymocyte Selection–Associated High-Mobility Group Box Family Member 4 (TOX4) as a factor recruited to both laser-induced DNA damage and endonuclease-induced DNA double-strand breaks. Depletion of TOX4 leads to accumulation of DNA damage, which is epistatic to DNA-PKcs. Consistently, TOX4 depletion substantially reduces NHEJ efficiency measured using both intrachromosomal and extrachromosomal repair assays. Our proteomic and biochemical analyses reveal TOX4 association with DNA-PK that is required for DNA-PKcs activation. Furthermore, we show that TOX4 coordinates with phosphatase 1 nuclear-targeting subunit in NHEJ. Phosphatase 1 nuclear-targeting subunit, previously shown to protect DNA-PKcs phosphorylation from protein phosphatase 1–mediated dephosphorylation, binds DNA-PK in a TOX4-dependent manner. In line with its role in DNA repair, TOX4 emerges as a promising target for anticancer drug development, and its targeting enhances tumor cell sensitivity to DNA damage in head and neck cancer and other malignancies.

The genome of a cell faces constant challenges from both exogenous and endogenous DNA-damaging agents, such as radiation, genotoxic chemicals, free radicals, and replication stress. These threats pose a significant risk to genomic integrity and can lead to the potential development of various diseases, including developmental defects, immune deficiency, and cancer (1). In accordance, to avoid genomic instability, cells have evolved a sophisticated DNA damage response system, to sense DNA damage and orchestrate DNA repair (2). Among the various types of DNA lesions, double-strand breaks (DSBs) represent one of the most severe, capable of causing catastrophic genomic damage. Cells employ two primary pathways for repairing DSBs: homologous recombination and nonhomologous end joining (NHEJ) (3). NHEJ, by directly ligating broken DSB ends without requiring a homologous strand as repair template, functions throughout the cell cycle and is a vital mechanism for genome maintenance (4, 5).

In NHEJ, broken DNA ends are recognized and bound by KU70/80 heterodimer, a component of the DNA-dependent protein kinase complex. The KU proteins then recruit DNA-dependent protein kinase catalytic subunit (DNA-PKcs), leading to its activation. The assembly of DNA-PK at DSB ends serves as a platform to recruit artemis, DNA ligase IV, and other NHEJ factors that are involved in end processing and ligation (6, 7, 8). Upon activation, DNA-PKcs phosphorylates numerous substrates, including XRCC4, artemis, and most importantly, DNA-PKcs itself. Mounting evidence revealed that DNA-PKcs phosphorylation, mediated by itself and other kinases, controls DNA-PKcs activity and configuration, and the subsequent end processing, in a sophisticated and site-specific manner (9, 10, 11).

In addition to the core mechanism of NHEJ, recent studies have characterized a number of accessory factors as modulators of NHEJ. For example, paralog of XRCC4 and XLF binds KU proteins, stabilizes the synapsis of DNA ends, and shows functional redundancy with XLF (12). Modulator of retroviral infection/cell cycle regulator of NHEJ (CYREN) fine tunes NHEJ in a cell cycle–dependent manner (13). Transactivation response of DNA-binding protein of 43 kDa facilitates the recruitment of ligase IV-XRCC4 (14). RNase H2 removes the ribonucleotides inserted by DNA polymerase μ during NHEJ (15). Our previous study showed that phosphatase 1 nuclear targeting subunit (PNUTS), a regulatory subunit of protein phosphatase 1 (PP1), is recruited to DNA damage sites to promote NHEJ (16). Multiple *in vitro* and cellular analyses indicated that dephosphorylation of DNA-PKcs by phosphatases at the inhibitory sites of DNA-PKcs triggers its activation (17, 18, 19). On the other hand, PNUTS associates with the DNA-PK complex to prevent PP1 from dephosphorylating DNA-PKcs at the DNA damage–induced activation sites, such as Ser-2056 (16).

Thymocyte Selection–Associated High-Mobility Group (HMG) Box Factor (TOX) is a family of evolutionarily conserved DNA-binding proteins, comprising TOX1–4 in mammalian cells. TOX genes encode an HMG motif with DNA-binding affinities, allowing them to regulate chromatin structure and gene transcription (20). TOX genes are known to play important roles in the immune system, such as T-cell development (21). Recent studies have also connected TOX expression to diverse types of human tumors, in association with tumor progression (22, 23). Interestingly, TOX1 was shown to bind and suppress KU proteins, thereby reducing the DNA damage recruitment of KU proteins and inhibiting NHEJ (24).

In this study, we show that a member of the TOX family, TOX4, also known as Lcp1 (Langerhans cell protein 1), was recruited to DNA DSB sites. In contrast to TOX1, TOX4 binds DNA-PK complex and promotes NHEJ. TOX4 coordinates with PNUTS in mediating DNA-PKcs activation and NHEJ.

## Results

### TOX4 is recruited to DNA damage sites

**Figure 1:**
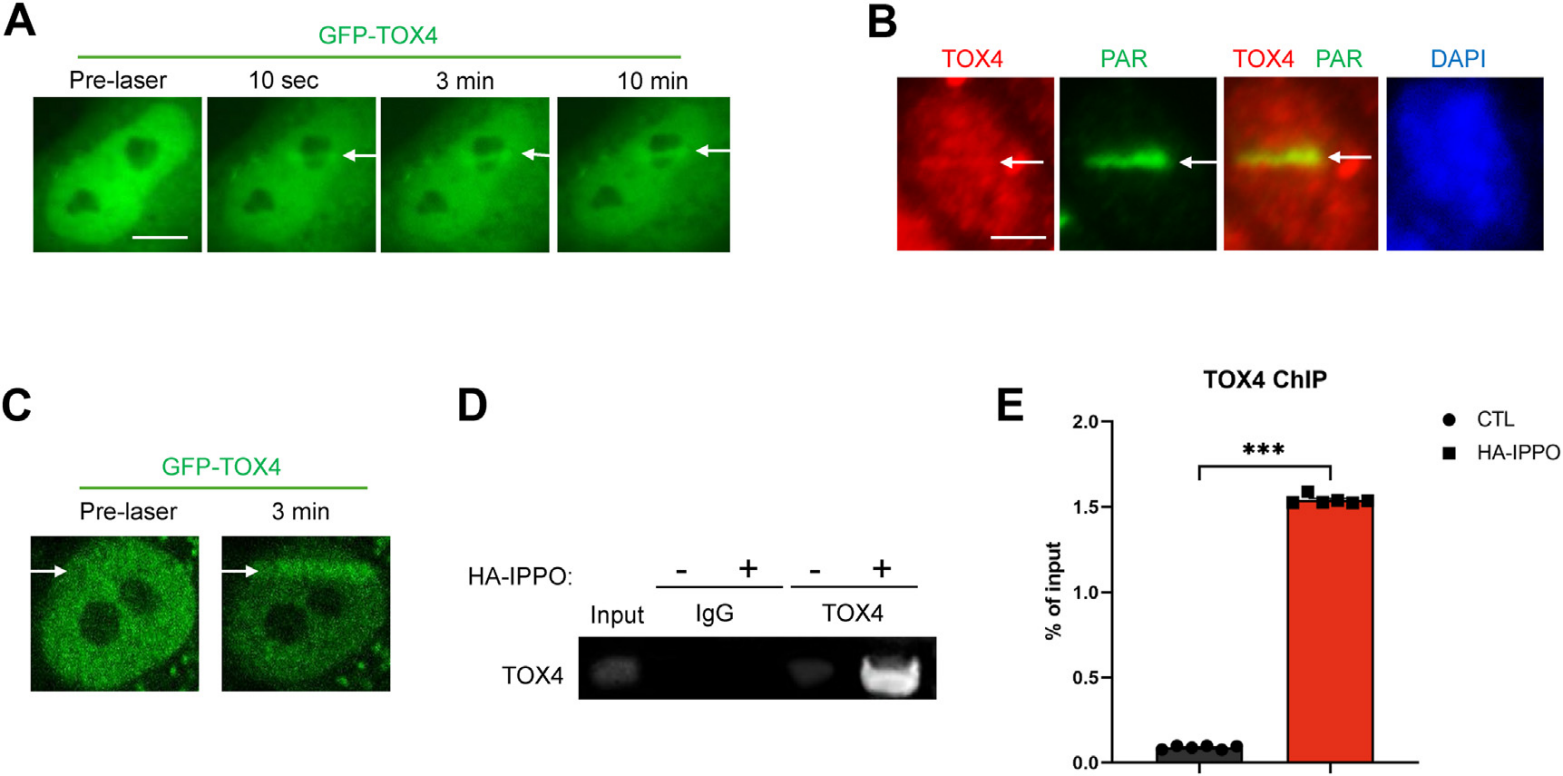
**The recruitment of TOX4 to DNA damage sites.***A*, GFP-TOX4–expressing HeLa cells were microirradiated with laser (405 nm, system 1), as described in the Experimental procedures section. The recruitment of GFP-TOX4 to laser-induced DNA damage is shown. Scale bar represents 5 μm. *B*, HeLa cells were treated with laser microirradiation, as in *A*, and analyzed by immunofluorescence for TOX4 and poly-ADP-ribose (PAR, as a marker of DNA damage induction). The area of laser microirradiation is denoted by the *white arrow*. Scale bar represents 5 μm. *C*, GFP-TOX4–expressing HeLa cells were microirradiated with laser (405 nm, system 2), as described in the Experimental procedures section. The prelaser and recruitment of GFP-TOX4 to laser-induced DNA damage after 3 min are shown. *D*, HeLa cells were transfected with or without HA-I-PpoI for 1 day, followed by chromatin immunoprecipitation, as described in the Experimental procedures section. TOX4 antibody or control IgG were used for immunoprecipitation, and a pair of the PPO-ribosomal DNA primers were used for PCR amplification. *E*, quantification of the chromatin immunoprecipitation results, as in *C*, statistical significance was analyzed using an unpaired two-tailed Student *t* test (∗∗∗*p* < 0.001). TOX4, Thymocyte Selection–Associated High-Mobility Group Box Family Member 4.

TOX4 was pulled down from cell nuclear extracts with platinum-induced DNA adducts (25), and TOX4-associated protein, PNUTS, was shown to mediate DNA repair (16, 26, 27). These findings prompted us to reveal the potential involvement of TOX4 in DNA repair. Interestingly, TOX4 was recruited to laser-induced DNA damage in cells, using two distinct laser microirradiation systems (Figs. 1, *A*–*C* and S1). The enrichment of TOX4 to DNA damage sites occurred within seconds, suggesting it as an early responder of DNA damage (Fig. 1*A* and Movie S1). Laser microirradiation induces mixed types of DNA damage, and the induction of DSB was confirmed by the recruitment of KU80 and Pol lambda (Fig. S1, *A* and *C*). Furthermore, we employed I-PpoI endonuclease to specifically induce genomic DNA DSBs. I-PpoI endonuclease introduces DSBs in repetitive 28S ribosomal DNA and other genomic loci (28). The subsequent chromatin immunoprecipitation analysis confirmed the recruitment of TOX4 to DSB sites, reaching over 40-fold enrichment after I-PpoI induction (Fig. 1, *D* and *E*). These data indicate that TOX4 plays a role in the cellular responses to DNA damage, especially DNA DSBs.

### TOX4 mediates DNA DSB repair *via* NHEJ

**Figure 2:**
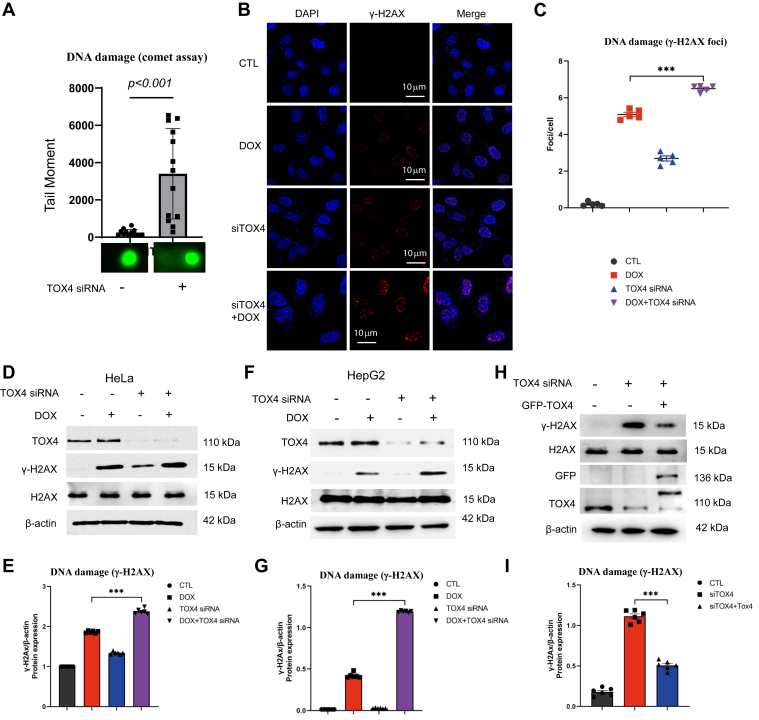
**TOX4 depletion causes DNA damage accumulation.***A*, the neutral comet assay was performed in HeLa cells with control or TOX4 siRNA, as described in the Experimental procedures section. Tail moment = %DNA tail × tail length (μm) was quantified, and shown as mean ± SEM, with representative comet images shown. Two-tailed unpaired Student *t* test was performed (N > 20, *p* < 0.001). *B*, HeLa cells were treated with doxorubicin (DOX, 5 μM, 4 h) and TOX4 siRNA, as indicated. Cells were analyzed by immunofluorescence, and representative images of γ-H2AX (*red*) and DAPI (*blue*) are shown. Scale bar represents 10 μm. *C*, cells were treated as in *B*. Representative fluorescence images (*red*, γ-H2AX; *blue*, DAPI) and quantification of γ-H2AX immunostaining in cells using ImageJ. Statistical significance was analyzed using GraphPad Prism. *D*, HeLa cells were treated with TOX4 siRNA or DOX, as in *B*. The cell lysates were analyzed by immunoblotting for γ-H2AX, H2AX, TOX4, and β-actin. *E*, corresponding densitometric analyses of γ-H2AX/β-actin, in *D*, were shown. Statistical significance was analyzed using an unpaired two-tailed Student *t* test (∗∗∗*p* < 0.001). *F*, HepG2 cells were treated with TOX4 siRNA or DOX, as in *B*. The cell lysates were analyzed by immunoblotting for γ-H2AX, H2AX, TOX4, and β-actin. *G*, corresponding densitometric analyses of γ-H2AX/β-actin, in *F*, are shown. Statistical significance was analyzed using an unpaired two-tailed Student *t* test (∗∗∗*p* < 0.001). *H*, HeLa cells were transfected with TOX4 siRNA, and siRNA-resistant GFP-TOX4, as indicated. The cell lysates were analyzed by immunoblotting for γ-H2AX, H2AX, TOX4, GFP, and β-actin. *I*, corresponding densitometric analyses of γ-H2AX/β-actin, in *H*, are shown. Statistical significance was analyzed using an unpaired two-tailed Student *t* test (∗∗∗*p* < 0.001). DAPI, 4',6-diamidino-2-phenylindole; TOX4, Thymocyte Selection–Associated High-Mobility Group Box Family Member 4.

We observed that depletion of TOX4 by siRNA induced accumulation of endogenous DNA damage in HeLa cells, as measured by single-cell electrophoresis (comet assay, Fig. 2*A*). The induction of DNA damage was also labeled by γ-H2AX, a phosphorylated form of histone H2AX that is commonly used as a marker of DNA damage, particularly DSBs (29, 30). The levels of phosphorylated histone H2AX significantly increased after TOX4 depletion, as shown by both immunofluorescent detection of γ-H2AX foci (Fig. 2, *B* and *C*) and immunoblotting (IB) of γ-H2AX (Figs. 2, *D* and *E* and S2*A*). Combining TOX4 siRNA with doxorubicin, a DNA-damaging drug used in chemotherapy, led to further induction of γ-H2AX in both immunofluorescent and IB analyses (Fig. 2, *B*–*E*). Consistent findings were also shown in HepG2 cell line (Fig. 2, *F* and *G*). Re-expression of RNAi-resistant TOX4 suppressed the induction of γ-H2AX, confirming the specific effect of TOX4 knockdown, in both HeLa (Fig. 2, *H* and *I*) and HepG2 cell line (Fig. S2, *B* and *C*).

**Figure 3:**
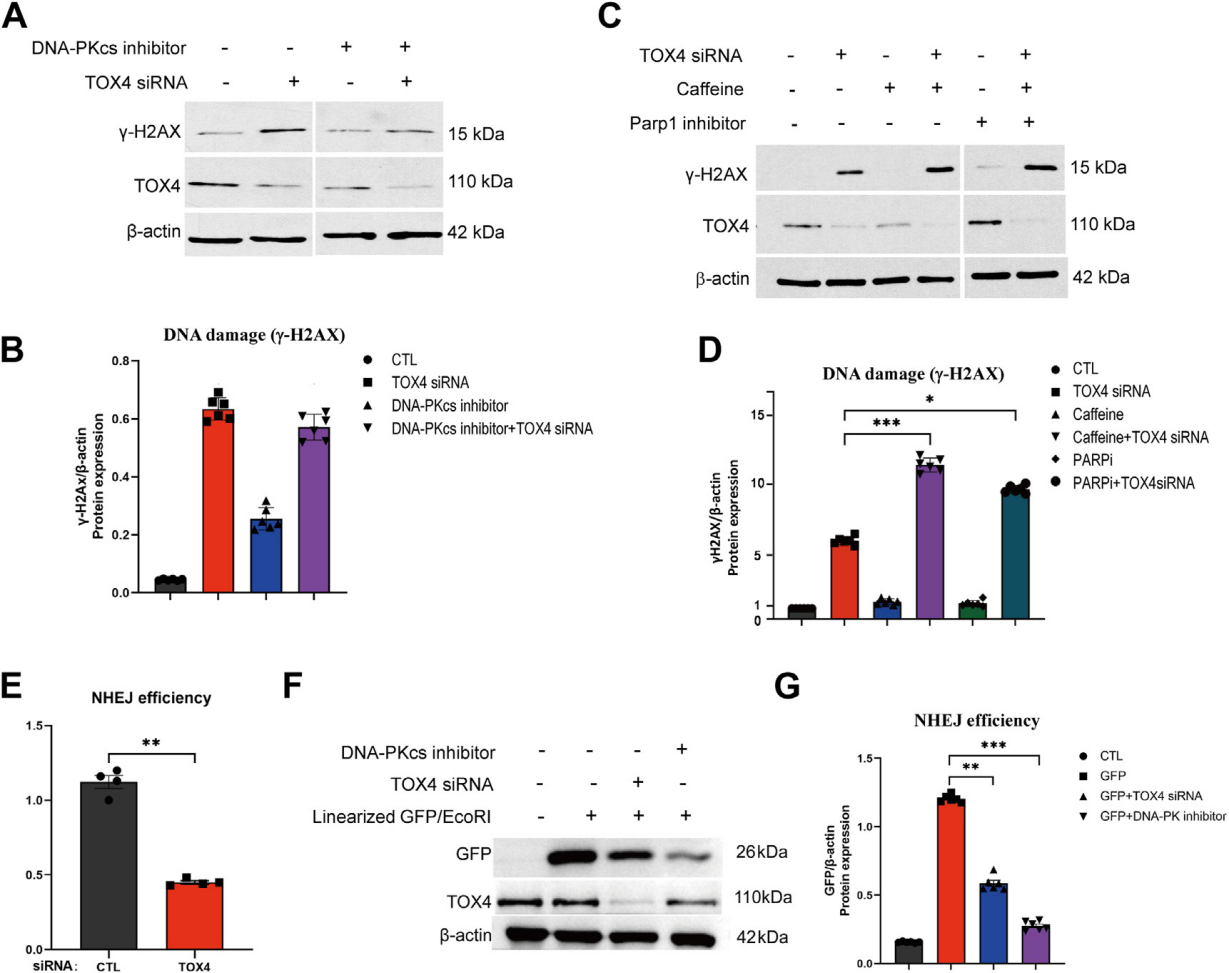
**TOX4 mediates DNA repair *via* NHEJ.***A*, HeLa cells were treated with TOX4 siRNA and DNA-PKcs inhibitor NU7026 (10 μM) for 24 h, as indicated. The cell lysates were analyzed by immunoblotting for γ-H2AX, TOX4, and β-actin. *B*, corresponding densitometric analyses of γ-H2AX/β-actin, in I, were shown. *C*, HeLa cells were treated with TOX4 siRNA, caffeine (2 mM), and PARP inhibitor olaparib (10 μM) for 24 h, as indicated. The cell lysates were analyzed by immunoblotting for γ-H2AX, TOX4, and β-actin. *D*, corresponding densitometric analyses of γ-H2AX/β-actin, in *K*, are shown. Statistical significance was analyzed using an unpaired two-tailed Student *t* test (∗*p* < 0.05, ∗∗∗*p* < 0.001). *E*, NHEJ efficiency was measured using a chromosome-integrated, I-SceI-based NHEJ reporter (U2OS-EJ5) in which NHEJ repair leads to GFP expression. Cells were transfected with control or TOX4 siRNA, and DNA repair was measured by immunoblotting of GFP expression in relative to β-actin. The mean values and SDs, calculated from four independent experiments, are shown. *F*, an extrachromosomal NHEJ reporter was designed, as described in the Experimental procedures section, using pEGFP-N vector linearized by EcoRI endonuclease. Upon transfection into HeLa cells, the repair of this vector resulted in GFP expression. Cells were treated with TOX4 siRNA or DNA-PKcs inhibitor NU7026, as indicated. Immunoblotting of GFP, TOX4, and β-actin is shown. *G*, NHEJ repair assay, as in N, was quantified for the ratio of GFP/β-actin expression. Statistical significance was determined using an unpaired two-tailed Student *t* test (∗∗*p* < 0.01, ∗∗∗*p* < 0.001). DNA-PKcs, DNA-dependent protein kinase catalytic subunit; NHEJ, nonhomologous end joining; TOX4, Thymocyte Selection–Associated High-Mobility Group Box Family Member 4.

Collectively, our results suggested that TOX4 functions in DNA repair, such that its depletion causes DNA damage accumulation. Thus, we sought out to indicate TOX4 in specific repair pathways. Interestingly, the effect of TOX4 depletion on γ-H2AX induction was largely diminished with the DNA-PKcs inhibitor NU7026 (Fig. 3, *A* and *B*) but not with that of ATM/ATR or PARP (Figs. 3, *C* and *D* and S2*D*). The epistatic relationship between TOX4 and DNA-PKcs suggested the involvement of TOX4 in NHEJ. Indeed, using a well-established intrachromosomal, I-SceI-based NHEJ assay (31), we showed that TOX4 depletion significantly reduced NHEJ (Fig. 3*E*). The same conclusion was also reached in an extrachromosomal NHEJ assay using linearized plasmid DNA expressing GFP in both HeLa (Fig. 3, *F* and *G*) and HepG2 cell lines (Fig. S2, *E* and *F*). This extrachromosomal NHEJ assay was validated using two DNA-PKcs inhibitors, which demonstrated the expected inhibitory effects on NHEJ (Figs. 3, *F* and *G* and S2, *E*–*H*). Compared with DNA-PKcs inhibition, TOX4 depletion was generally less effective in suppressing NHEJ, potentially because of the partial nature of its depletion or the involvement of redundant pathways (Figs. 3, *F* and *G* and S2, *E*–*H*). Moreover, combining TOX4 depletion with DNA-PKcs inhibitor ADZ7648 did not significantly further enhance the suppression of NHEJ compared with ADZ27648 treatment alone (Fig. S2, *G* and *H*).

### TOX4 associates with DNA-PK complex and facilitates DNA-PKcs activation

**Figure 4:**
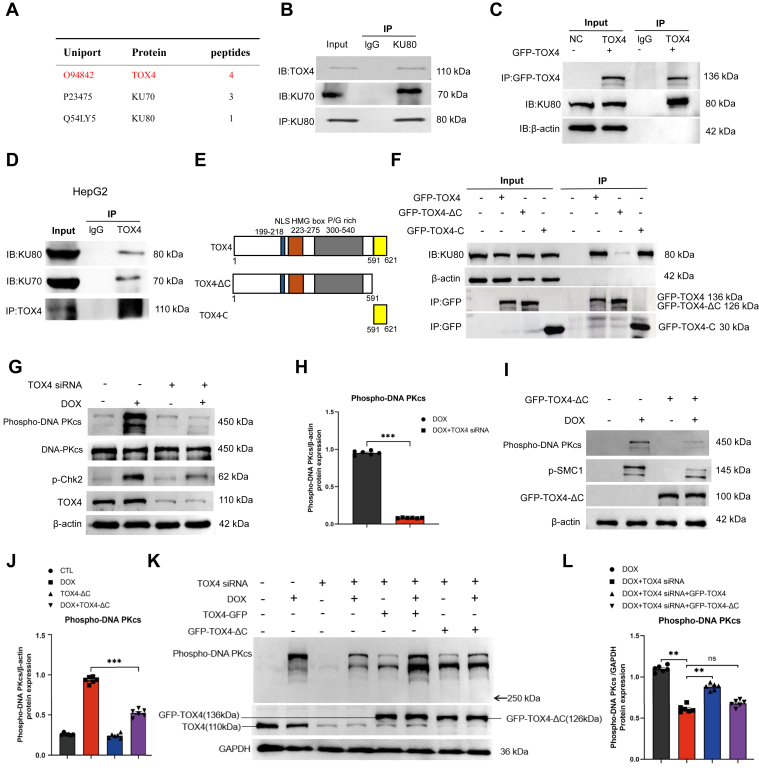
**TOX4 binds KU proteins and mediates DNA-PKcs activation.***A*, TOX4 immunoprecipitation (IP) was performed in HeLa cell lysates, and the IP project was subjected to mass spectrometric identification. KU70 and KU80 were among the identified proteins, as shown with the number of peptides. A control IP using IgG did not recover any peptides of TOX4, KU70, or KU80. The specific binding proteins of TOX4, identified in this proteomic study, are shown in Table S1. *B*, KU80 IP was performed in HeLa cell lysates. The lysate input at 20%, control (ctr) IP with blank beads and KU80 IP products were analyzed by immunoblotting for TOX4, KU70, and KU80. *C*, GFP-TOX4 was expressed in HeLa cells, and GFP IP was performed. The lysate input at 20%, control IP, and GFP IP products was analyzed by immunoblotting for TOX4, KU80, and β-actin. *D*, TOX4 IP was performed in HepG2 cell lysates. The lysate input at 20% control (ctr) IP with blank beads and TOX4 IP products were analyzed by immunoblotting for TOX4, KU70, and KU80. *E*, the schematic diagram of TOX4 mutants generated in this study. *F*, full-length and two segments of TOX4 (ΔC: aa 1–591; C: aa 591–621) were tagged with GFP and expressed in HeLa cells. GFP IP was performed in cells expressing these proteins or control cells. The lysate input at 20% and GFP IP products were analyzed by immunoblotting for GFP, β-actin, and KU80. *G*, HeLa cells were treated with TOX4 siRNA and doxorubicin (DOX) (5 μM) for 4 h, as indicated. The cell lysates were analyzed by immunoblotting for phospho-DNA-PKcs Ser-2056, DNA-PKcs, Chk2 phospho-Thr-68, TOX4, and β-actin. *H*, cells were analyzed as in *G*. Corresponding densitometric measurement of phospho-DNA-PKcs Ser-2056 corrected by β-actin was shown. Statistical significance was analyzed using an unpaired two-tailed Student *t* test (∗∗∗*p* < 0.001). *I*, HeLa cells were transfected with TOX4-ΔC segment and treated with DOX, as indicated. The cell lysates were analyzed by immunoblotting for DNA-PK phospho-Ser-2056, Smc1 phospho-Ser-957, TOX4, and β-actin. *J*, corresponding densitometric analyses of phosphor-DNA-PKcs Ser-2056/β-actin, in *I*, are shown. Statistical significance was analyzed using an unpaired two-tailed Student *t* test (∗∗∗*p* < 0.001). *K*, HeLa cells were transfected with TOX4 siRNA, TOX4-ΔC, and TOX4-WT, and treated with DOX, as indicated. The cell lysates were analyzed by immunoblotting for DNA-PKcs phospho-Ser-2056, TOX4, and GAPDH. *L*, corresponding densitometric analyses of phospho-DNA-PKcs Ser-2056/β-actin in *J* are shown. Statistical significance was determined using an unpaired two-tailed Student *t* test (∗∗*p* < 0.01, ns: *p* > 0.05). DNA-PKcs, DNA-dependent protein kinase catalytic subunit; TOX4, Thymocyte Selection–Associated High-Mobility Group Box Family Member 4.

We performed a proteomic analysis to identify proteins associated with TOX4. Consistent with the role of TOX4 in NHEJ, we identified KU70 and KU80 as TOX4-associated proteins (Fig. 4*A*). We subsequently confirmed the TOX4 and KU80 association by reciprocal coimmunoprecipitation in HeLa cell line (Fig. 4, *B* and *C*) and HepG2 cell line (Fig. 4*D*). The association between TOX4 and KU80 remained intact despite nuclease treatment, suggesting that it is not DNA mediated (Fig. S3*A*). Next, we sought to delineate the motif(s) of TOX4 that mediates the KU80 association (Fig. 4*E*). We observed that both the full-length and C terminus segments of TOX4 bound KU80, whereas the C terminus deleted segment exhibited substantially reduced KU80 association (Fig. 4*F*).

Next, we investigated DNA-PKcs activation indicated by its autophosphorylation at Ser-2056. Interestingly, TOX4 depletion abolished DNA-PKcs autophosphorylation induced by doxorubicin in HeLa cell line (Fig. 4, *G* and *H*) and HepG2 cell line (Fig. S3, *B* and *C*). Similar results were also shown with treatments using bleomycin (Fig. S3, *D* and *E*) and cisplatin (Fig. S3, *F* and *G*). TOX4 did not alter the protein level of DNA-PKcs (Figs. 4*G* and S3, *D* and *F*). Moreover, expression of TOX4-ΔC deficient of KU70/80 binding exhibited a dominant-negative effect in suppressing doxorubicin-induced DNA-PKcs autophosphorylation (Fig. 4, *I* and *J*) and cisplatin-induced DNA-PKcs autophosphorylation (Fig. S3*H*). DNA-PKcs autophosphorylation decreased by TOX4 depletion was rescued by re-expression of WT TOX4 but not TOX4-ΔC (Fig. 4, *K* and *L*), confirming that TOX4 binding to DNA-PK is indispensable for efficient activation of DNA-PKcs after DNA damage.

### TOX4 coordinates with PNUTS in NHEJ

**Figure 5:**
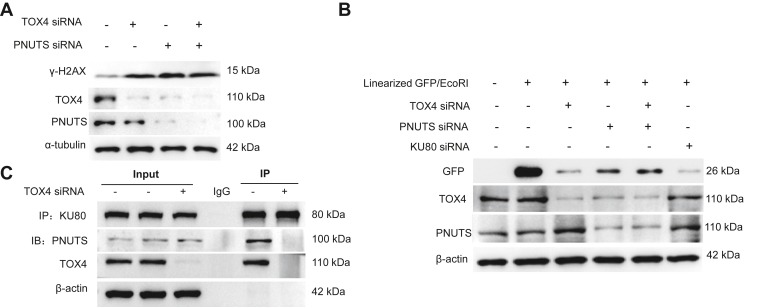
**TOX4 and PNUTS coordinate in NHEJ.***A*, HeLa cells were treated with siRNA targeting TOX4 or PNUTS, as indicated, for 24 h. The cell lysates were analyzed by immunoblotting for γ-H2AX, TOX4, PNUTS, and tubulin. *B*, NHEJ repair efficiency was measured in HeLa cells using the linearized GFP vector, as in Figure 2*N*. Cells were treated with or without siRNA targeting TOX4, PNUTS, or KU80, as indicated. Immunoblots of GFP, TOX4, PNUTS, and β-actin are shown. *C*, Ku80 IP was performed in HeLa cells treated with control or TOX4 siRNA. The lysate input at 20%, control (ctr) IP with blank beads, and KU80 IP products were analyzed by immunoblotting for PNUTS, TOX4, KU80, and β-actin. NHEJ, nonhomologous end joining; PNUTS, phosphatase 1 nuclear targeting subunit; TOX4, Thymocyte Selection–Associated High-Mobility Group Box Family Member 4.

As TOX4 and PNUTS are known to be associated proteins, we asked if they coordinate in NHEJ. Interestingly, PNUTS depletion also reduced TOX4, whereas TOX4 siRNA did not affect PNUTS (Fig. 5*A*). Presumably, TOX4 stabilization is dependent on PNUTS but not *vice versa*. Both TOX4 and PNUTS depletion resulted in increased levels of γ-H2AX (Fig. 5*A*) and reduced NHEJ (Fig. 5*B*). Double deletion of TOX4 and PNUTS did not cause further effects from single depletion of TOX4 or PNUTS (Fig. 5, *A* and *B*), suggesting their coordinative involvement in DNA repair. Along this line, TOX4 and PNUTS dual depletion did not further increase γ-H2AX in TOX-treated cells (Fig. S4*A*). We have shown previously that PNUTS associated with KU proteins to promote NHEJ (16). The association between KU80 and PNUTS was disrupted by TOX4 depletion (Fig. 5*C*), indicating that TOX4 bridges the KU80 and PNUTS association. PNUTS was also shown to promote PARP1 activation after DNA damage (27). However, TOX4 was not required for the induction of PARylation after DNA damage (Fig. S4*B*), suggesting that this function of PNUTS is achieved independent of TOX4.

### TOX4 is a potentially effective anticancer drug target to enhance DNA damage sensitivity

**Figure 6:**
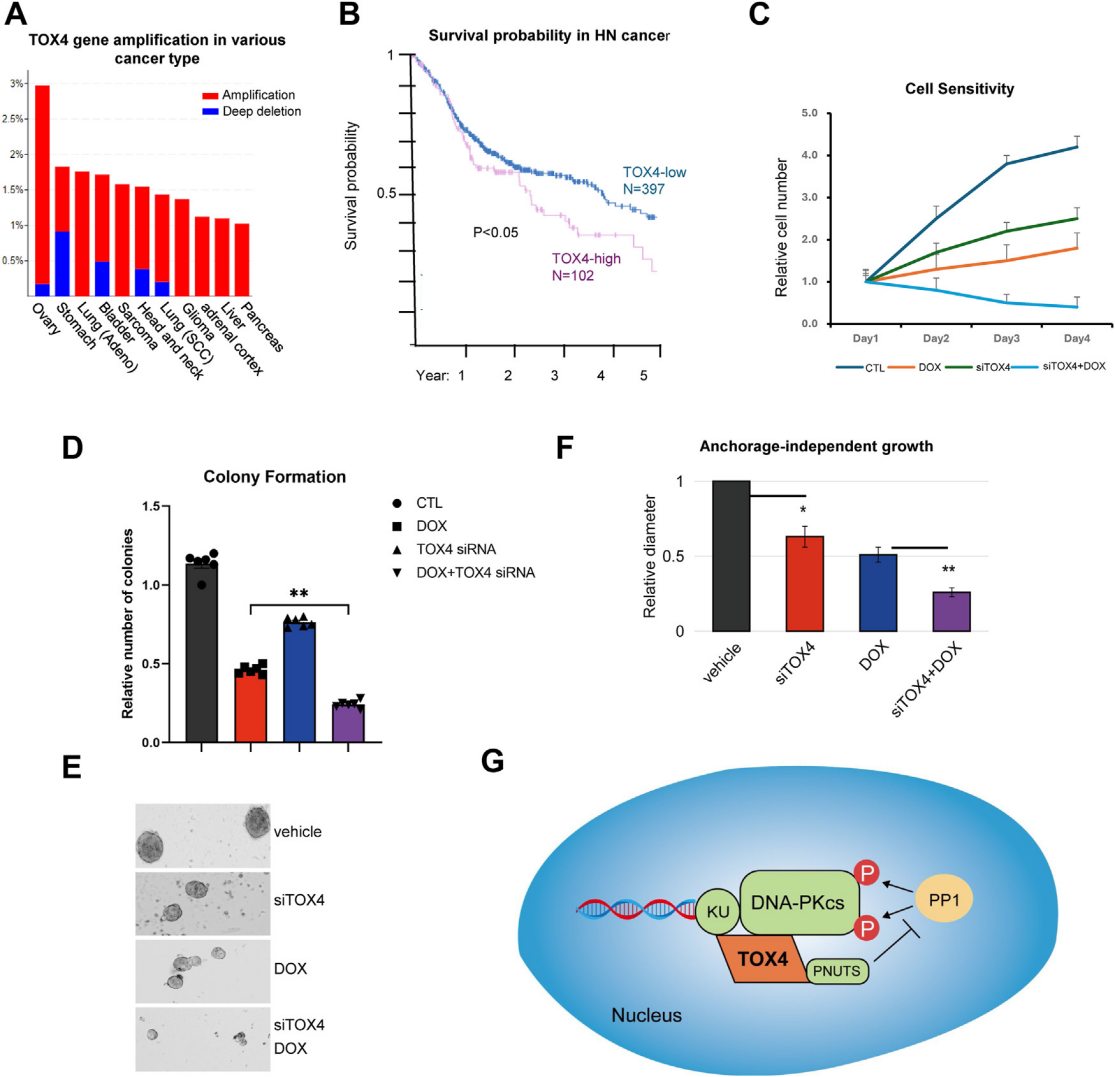
**TOX4 promotes treatment resistance in cancer.***A*, the Cancer Genome Atlas database analysis of TOX4 gene amplification in various types of cancer and representative of amplification (*red*) and deep deletion (*blue*) are shown. *B*, Kaplan–Meier survival analysis of head and neck cancer was performed, in groups with high or low expression of TOX4. *C*, HeLa cells were treated with control or TOX4 siRNA at day 0, incubated with doxorubicin (DOX) at day 1 and maintained in culture for 3 days. Cell viability was determined in each day and normalized to that of day 1. The mean value and SD were calculated from three independent experiments. *D*, the clonogenic assay was performed as described in the Experimental procedures section. The numbers of colonies were normalized to untreated control. The mean value and SD were calculated from three independent experiments. Statistical significance was analyzed using an unpaired two-tailed Student *t* test. *E*, UM-SCC-38 cells with or without TOX4 siRNA and DOX were cultured in nonadhesive dishes for anchorage-independent growth. Spheroid growth was imaged and shown. *F*, spheroid size, as in *E*, was measured, and shown. Statistical significance was determined using an unpaired two-tailed Student *t* test (∗*p* < 0.05, ∗∗*p* < 0.01). *G*, schematic diagram summarizing the role of TOX4 in promoting DNA-PKcs activation and NHEJ. The subsequent accumulation of TOX4, at upregulated levels several hours after DNA damage. TOX4 bound both PNUTS and DNA-PK, and was required for the association of PNUTS with DNA-PK, which modulates DNA-PKcs activation after DNA damage. DNA-PKcs, DNA-dependent protein kinase catalytic subunit; NHEJ, nonhomologous end joining; PNUTS, phosphatase 1 nuclear targeting subunit; TOX4, Thymocyte Selection–Associated High-Mobility Group Box Family Member 4.

DNA-damaging agents are often cornerstone treatment options for cancer. Accordingly, DNA repair is known to mediate treatment resistance in cancer, and its targeting has been proposed as a potentially effective strategy to overcome tumor resistance. Interestingly, our analysis of The Cancer Genome Atlas database uncovered amplification of TOX4 gene in 1 to 3% of various types of cancer, including those of ovary, stomach, lung, bladder, sarcoma, head and neck, brain, adrenal cortex, liver, and pancreas (Fig. 6*A*). In head and neck cancer, the expression level of TOX4 correlated with adverse survival probability (Fig. 6*B*); similar patterns of association between TOX4 expression and shorter patient survival were also seen in stomach cancer, urothelial cancer, and pancreatic cancer (Fig. S5, *A*–*C*). We further revealed that TOX4 depletion profoundly sensitized cells to doxorubicin (Fig. 6*C*). This phenotype was further confirmed in a colony formation assay (Fig. 6*D*). Because the anchorage-independent growth potential constitutes a physiologically important feature of tumor cells (32), we studied the impact of TOX4 depletion on the growth and treatment response of head and neck cancer cells in anchorage-independent spheroid culture. TOX4 depletion resulted in a moderate but significant reduction in spheroid number and size (Fig. 6, *E* and *F*), with even stronger effects observed when combined with doxorubicin treatment (Fig. 6, *E* and *F*).

## Discussion

As a member of the HMG-box protein family, TOX4 has been reportedly involved in DNA reprogramming, transcription modulation, and apoptosis (23, 33, 34). Here, we characterized a new role of TOX4 in the DNA damage response, particularly NHEJ. We presented novel evidence to show that TOX4 was recruited to sites of DNA damage, including DSBs, in cells. TOX4 depletion impaired NHEJ repair and led to accumulation of endogenous DNA damage. TOX4 bound KU proteins and facilitated DNA-PKcs activation after DNA damage. Thus, while TOX4 may indirectly impact DNA repair and damage responses by modulating chromatin and transcription, our findings highlight its direct recruitment to DNA damage sites, its association with DSB repair factors, and its role in facilitating DNA repair.

Our studies indicated that TOX4 and PNUTS coordinated in promoting DNA-PKcs activation and NHEJ. PNUTS was previously shown to protect DNA-PKcs Ser-2056 phosphorylation from PP1-mediated dephosphorylation. TOX4 bound both PNUTS and DNA-PK and was required for the association of PNUTS with DNA-PK (Fig. 6*G*).

These findings add to the emerging understanding of DNA-PKcs regulation *via* phosphorylation. As a large protein with diverse functional domains, DNA-PKcs is controlled in its molecular configuration and kinase activation by sophisticated mechanisms. Multiple domains of DNA-PKcs contain self-inhibitory phosphorylation sites, in line with the findings that several serine/threonine PPs act as activators of DNA-PKcs (16, 17, 18, 19). On the other hand, DNA damage induces the phosphorylation of DNA-PKcs at numerous sites, mediated by DNA-PKcs itself and ATM. These phosphorylation events are required for NHEJ, by promoting DNA-PKcs activation and orchestrating the molecular dynamics of DNA-PK at DSB ends. It is plausible that this fashion of complex and site-specific phosphoregulation is achieved through additional accessory factors that bind DNA-PK and modulate the action of PPs. Indeed, our findings suggest a model that TOX4 recruits PNUTS to DNA-PK to prevent PP1-mediated dephosphorylation of DNA-PKcs Ser-2056 (Fig. 6). Notably, in addition to PP1, numerous other PPs, including PP2A, PP5, and PP6, were implicated in DNA-PKcs regulation (17, 18, 19). Our previous study suggested the association of these phosphatases with distinct domains of DNA-PKcs (16). Thus, future studies are needed to better understand how these phosphatases are regulated, likely with the involvement of additional accessory factors, to result in dynamic and site-specific phosphoregulation of DNA-PKcs.

We discovered that the protein stabilization of TOX4 requires PNUTS, indicating their intimate relationship. Interestingly, the PNUTS–TOX4 complex has been implicated in various cellular processes. For example, PNUTS–TOX4–WDR82 complexes with PP1 to modulate gene expression. While PNUTS restrains the action of PP1 toward other substrates, PNUTS–TOX4–WDR82 directs PP1 to dephosphorylate components of the RNA polymerase II machinery, to regulate gene expression, control the speed of RNA polymerase II, and prevent transcription and replication collisions (35, 36).

The herein reported role of TOX4 in NHEJ differs from the inhibitory effect of TOX1 in DNA repair, underscoring the distinct and potentially counteracting roles of TOX genes. Although TOX1 has been well established in the development and function of T cells, the functions of other TOX genes are relatively less understood (37). TOX2, implicated in the immune system like TOX1, has also been suggested to play a role in neuronal differentiation and maturation (38, 39). TOX3, also known as TNRC9, may function in neuronal development and function, reminiscent of TOX2 (40). Furthermore, TOX3 has been increasingly associated with human cancer, particularly breast cancer (41). It is intriguing that TOX1 and TOX4 are likely to have opposing functions in NHEJ. While TOX1 bound KU proteins to suppress the recruitment of DNA-PK to DNA damage sites, TOX4 is itself recruited to DSB sites, facilitating DNA-PK activation and NHEJ. As such, TOX1 and TOX4 potentially compete to fine tune DNA repair *via* NHEJ. Along this line, we indeed observed the opposing effect of TOX1 and TOX4 depletion on DNA-PKcs activation (Fig. S6, *A* and *B*).

Targeting NHEJ has been proposed in cancer therapy, as a potentially effective way to improve the treatment outcomes of radiation and DNA-damaging drugs. Conversely, upregulation of NHEJ factors may enable tumor cells to gain DNA damage resistance and evade treatments (42, 43). Clinical data illustrated the gene amplification and upregulation of TOX4 in various types of cancer, including head and neck cancer where TOX4 expression correlated with adverse patient survival. Consistent with the function of TOX4 in DNA repair, our study showed that targeting TOX4 in conjunction with DNA-damaging drugs exhibited enhanced tumor cell responses. Thus, future studies shall uncover if therapeutic intervention of TOX4 functions and interactions are of clinical potential for anticancer treatments, in combination with DNA damage or other anti–DNA repair agents.

## Experimental procedures

### Cell culture and treatment

The HeLA (human cervix carcinoma) and HepG2 (human hepatocellular carcinoma) cell lines were authenticated by American Type Culture Collection. U2OS-EJ5 (human osteosarcoma) and UM-SCC-38 (human head and neck cancer) cell lines were described as in our previous studies (44, 45). Cells were maintained in Dulbecco’s modified Eagle's medium (Hyclone) with 10% fetal bovine serum (Hyclone). Cell viability assays were performed as in our previous studies (46). Briefly, cells were incubated for 1 to 4 days. The numbers of viable cells were counted using a hemocytometer. To measure cell death, trypan blue staining was performed by mixing 0.4% trypan blue in PBS with cell suspension at a 1:10 ratio. 3D culture was performed using Nunclon Sphera ultra–low-attachment plates (ThermoFisher Scientific). SiRNAs targeting TOX4 (target sequence GGGCAUAGCCAGUUGACCATT or UGGUCAACUGGCUAUGCCCTT), PNUTS (target sequence UCUGACAAGUACAACCUU or GGCGGCUACAAACUUCUU), and TOX1 (target sequence GGGAAUGAAUCCUCACCUATT or UAGGUGAGGAUUCAUUCCCTT) were purchased from Integrated DNA Technologies and transfected into cells using a protocol recommended by the manufacturer. A nontargeting control or scramble siRNA was used as a control. H_2_O_2_ (CAS: 7728-84-1) and benzonase (CAS: 9025-65-4) were obtained from Sigma. Doxorubicin (CAS: 23214-92-8), bleomycin (CAS: 11056-06-7), cisplatin (CAS: 15663-27-1), AZD7648 (CAS: 2230820-11-6), VE822 (CAS: 1232416-25-9), KU55933 (CAS: 587871-16-9), and NU7026 (CAS: 154447-35-5) were purchased from Selleckchem.

### Immunoblotting

SDS-PAGE and IB were carried out as previously described (47), using the following antibodies: KU80 (A302-627A), γ-H2AX (A300-081A), PNUTS (A300-439A), and Smc1 phosphoS957 (A304-147A) antibodies from Bethyl Laboratories; GFP (sc-9996) and KU70 (sc-56129) antibodies from Santa Cruz Biotechnology; TOX4 (ab272576), Chk2 phospho-T387 (ab195783), PAR (ab14459), DNA-PKcs (ab70250), DNA-PKcs phospho-S2056 (ab18192) antibodies from Abcam; β-actin (#4970), tubulin (#2144), caspase-3 (#9662), and Chk2 phospho-T68 (#2661) antibodies from Cell Signaling Technology.

### I-PpoI assay and chromatin immunoprecipitation

The I-PpoI system was used to introduce DSBs in repetitive 28S ribosomal DNA and other genomic loci (28). Briefly, HeLa cells were transfected with control plasmid or pBABE-HA-ER-IPpoI (Addgene plasmid #32565, a gift from Michael Kastan (48)) using Lipofectamine (Invitrogen). To induce I-PpoI digestion, cells were treated with 5 μm 4-hydroxytamoxifen (Cayman Chemical Company) treatment for 24 h, prior to chromatin immunoprecipitation (ChIP). The ChIP assay was performed using the simple ChIP enzymatic chromatin IP kit (CST#9003), following the protocol provided by the manufacturer. Proteins in HeLa cells were crosslinked to DNA with fresh 1% formaldehyde solution for 10 min with briefly swirling at room temperature and then quenched with 10× glycine for 5 min. Cell nuclei were pelleted by repeating centrifugation, and DNA was digested to 150–900 bp with Micrococcal Nuclease #10011 and processed by sonication. The resulted cross-linked chromatin will be incubated with RNAse-A at 37 °C for 30 min and then with proteinase K at 65 °C for 2 h. DNA was purified, and its concentration was tested. IP was performed using the indicated primary antibodies, including positive control histone H3 or negative control normal rabbit IgG. The ChIP-grade protein G magnetic beads were added into each reaction for incubation for 2 h. The chromatin was eluted from the antibody/protein G beads, and crosslinks were reversed by NaCl and proteinase K. The final DNA products were purified and quantified by PCR analysis.

### Immunofluorescence and imaging

Immunofluorescence was performed as previously described (49). Briefly, cells were fixed in 3% formaldehyde with 0.1% Triton X-100, washed, and blocked in 10% goat serum in PBS. Primary antibodies were diluted in blocking buffer and incubated with the cells for 2 h. The cells were then incubated with secondary antibodies conjugated with Alexa Fluor 488/555 (Invitrogen; 1:2000 dilution) for 1 h. Imaging was performed using a Zeiss Axiovert 200M inverted fluorescence microscope.

Two independent systems were used for laser microirradiation. The first system utilized a 405 nm laser under the Zeiss Axiovert 200M Microscope with Marianas Software (Intelligent Imaging Innovations, Inc). Cells were not presensitized using this microirradiation system. In the other system, we performed 405 nm laser microirradiation after presensitization, under Zeiss 880 confocal laser scanning microscope. One hour prior to laser microirradiation, cells were pretreated with Hoechst 33342 (Invitrogen) at 1 μg/ml.

### Protein expression and immunoprecipitation

GFP-TOX4 was constructed by inserting human TOX4 to pEGFP-C1 (Clontech). TOX4-C (aa 592–619) and TOX4-ΔC (aa 1–591) was obtained by PCR amplification and inserted into the pEGFP-C1 vector for expression. Immunoprecipitation was performed using 300 μg of protein lysate along with 4 μg of TOX4 antibody, KU70 antibody (ab3108; Abcam), PNUTS antibody, or control rabbit IgG (Santa Cruz Biotechnology). Immunoprecipitation was completed in the presence of 30 to 40 μl of Dynabeads Protein G (Life Technologies) and incubated for 16 h at 4 °C in Cell Lysis Buffer (Cell Signaling Technology). Beads were then washed five times, and proteins were eluted using Laemmli buffer, at 95 °C for 10 min. Denatured proteins were loaded on a 4 to 20% gradient gel (Bio-Rad) for IB analysis.

### NHEJ assays

The NHEJ assay was performed in U2OS-EJ5 cells. Briefly, cells were seeded at 3 × 10^5^ cells per well in a 6-well plate for 24 h before siRNA treatment. After removing the siRNA, the cells were transfected with an expression vector of I-SceI endonuclease and cultured for 48 h. In this assay, GFP is expressed only after DSBs introduced by I-SceI endonuclease are repaired by NHEJ, and the levels of GFP expression, and loading control α-tubulin, were quantified by IB, using the National Institutes of Health ImageJ. The plasmid-based NHEJ assay was performed in HeLa cells. Briefly, pEGFP-N vector was linearized with EcoRI, gel purified, and transfected into HeLa cells. Cells were collected after 24 h incubation and analyzed by IB for GFP expression. The level of GFP was quantified and normalized to that of β-actin, using ImageJ.

### Neutral comet assay

HeLa cells were treated with control or TOX4 siRNA for 48 h and harvested by trypsinization and subsequently resuspended in PBS. Cells were mixed with freshly prepared 0.75% low-melting agarose at a 1:10 ratio (cell suspension to agarose). The mixture was pipetted onto slides precoated with 1% low-melting agarose. Slides were incubated for 1 h on ice in lysis buffer (3.5 M NaCl, 100 mM EDTA, 100 mM Tris base, 8 g NaOH [pH 10], 1% Triton X-100, and 10% dimethyl sulfoxide). Slides were cleaned for 30 min at 4 °C in 1× neutral electrophoresis buffer (prepared from a 10× stock solution: 60.57 g Tris base, 204.12 g sodium acetate, diluted in 450 ml H_2_O, with pH adjusted to 9 using glacial acetic acid). The slides were placed in the electrophoresis tank containing 1× neutral electrophoresis buffer, covered with an overlay, and subjected to electrophoresis at 100 V for 1 h at 4 °C. Following electrophoresis, slides were drained and immersed in DNA precipitation solution (7.5 M ammonium acetate dissolved in water and 95% ethanol) for 30 min at room temperature, followed by immersion in 70% ethanol for 5 min. Slides were allowed to air dry at room temperature for 10 min. Each slide was stained with 50 μl of diluted SYBR Gold (1:10,000 dilution) and incubated for 30 min. Imaging was performed under a fluorescent microscope (EVOS M5000), and quantification was carried out using ImageJ software.

### Statistical analysis

Data were expressed as means ± SD of at least three independent experiments. Statistical evaluation of the data was performed using the Student *t* test (and nonparametric tests) and one-way ANOVA (and nonparametric or mixed). The differences were statistically significant at *p* < 0.05 in the analytical treatment of the data. GraphPad Prism software (version 8.0.1) was used to process the statistical analysis.

## Data availability

All representative data are contained within the article and in the supporting information.

## Supporting information

This article contains supporting information.

## Conflict of interest

The authors declare that they have no conflicts of interest with the contents of this article.

## Supporting information

Sup Materials A2

Tox4 mass spec
