# Supplementary material for: TOX High-Mobility Group Box Family Member 4 promotes DNA double-strand break repair via nonhomologous end joining
Source: J Biol Chem. 2025 May 4;301(6):110174. doi: 10.1016/j.jbc.2025.110174 (PMC12166427; doi:10.1016/j.jbc.2025.110174)
Supplement: Sup Materials A2 [file mmc1.pdf]

**Figure S1. The recruitment of TOX4 to DNA damage sites. A.** GFP-KU70 and GFP-Pol lambda expressing HeLa cells were micro-irradiated with laser (405 nm, system 1), as described in Materials and Methods. The recruitment of GFP- KU70 and GFP-Pol lambda to laser-induced DNA damage in pre-laser and after 3 minutes were shown. The area of laser micro-irradiation is denoted an arrow. **B.** GFP-TOX4 expressing HeLa cells were micro-irradiated with laser (405 nm, system 2), as described in Materials and Methods. The recruitment of GFP-TOX4 to laser-induced DNA damage in pre-laser and after 3 minutes is shown. The area of laser micro-irradiation is denoted an arrow. **C.** GFP-Pol lambda expressing HeLa cells were micro-irradiated with laser (405 nm), as in panel B. The recruitment of GFP-Pol lambda to laser-induced DNA damage is shown. The area of laser micro-irradiation is denoted an arrow.

**Figure S2. TOX4 mediates DNA repair via NHEJ. A.** HeLa cells were transfected with TOX4 siRNA, and harvested at the indicated time points. Cells were harvested and analyzed by immunoblotting for γ-H2AX, H2AX, TOX4, GFP and β-actin. **B.** HepG2 cells were transfected with TOX4 siRNA, and siRNA-resistant GFP-TOX4, as indicated. The cell lysates were analyzed by immunoblotting for γ-H2AX, TOX4, GFP and β-actin. **C.** Corresponding densitometric analyses of γ-H2AX/β-actin, in panel B, were shown. Statistical significance was analyzed using an unpaired two-tailed Student's t test (***$p<0.001$). **D.** HeLa cells were treated with TOX4 siRNA, ATM inhibitor KU55933 (10 μM) and ATR inhibitor VE822 (5 μM), as indicated, for 24h. Cell lysates were analyzed by immunoblotting for γ-H2AX, γ-H2AX, TOX4 and β-actin. **E.** An extrachromosomal NHEJ reporter was designed using pEGFP-N vector linearized by EcoRI endonuclease. Upon transfection into HepG2 cells, the repair of this vector resulted in GFP expression. Cells were treated with TOX4 siRNA or DNA-PKcs inhibitor NU7026, as indicated. Immunoblotting of GFP, TOX4 and β-actin is shown. **F.** NHEJ repair assay, as in panel E, was quantified by the ratio of GFP/β-actin expression. Statistical significance was determined using an unpaired 2-tailed Student's t-test (**$p<0.01$,***$p<0.001$). **G.** The extrachromosomal NHEJ reporter, as in panel E, was transfected into HepG2 cells. Cells were treated with TOX4 siRNA or DNA-PKcs inhibitor AZD7648, as indicated. Immunoblotting of GFP, TOX4 and β-actin is shown. **H.** NHEJ repair assay, as in panel G, was quantified by the ratio of GFP/β-actin expression. Statistical significance was determined using an unpaired 2-tailed Student's t-test (**$p<0.01$,***$p<0.001$).

**Figure S3. TOX4 binds KU proteins and mediates DNA-PKcs activation. A.** HeLa cells lysates were treated with control and Benzonase (final concentration 200 units/mL) for 30 min, TOX4 IP was performed in cell lysates in both control group and Benzonase treated group. The lysate input at 20%, control (ctr) IP with blank beads, and TOX4 IP products were analyzed by immunoblotting for Ku80, TOX4, and β-actin. **B.** HepG2 cells were treated with TOX4 siRNA and doxorubicin (DOX, 5 μM) for 4 hr, as indicated. The cell lysates were analyzed by immunoblotting for phospho-DNA-PKcs Ser-2056 and β-actin. **C.** Cells were analyzed as in panel B. Corresponding densitometric measurement of phospho-DNA-PKcs

Ser-2056 corrected by β-actin was shown. Statistical significance was analyzed using an unpaired two-tailed Student's t test (**p<0.01). **D.** HeLa cells were treated with TOX4 siRNA and Bleomycin (5 μM) for 24 hr, as indicated. The cell lysates were analyzed by immunoblotting for phospho-DNA-PKcs Ser-2056, DNA-PKcs, γ-H2AX, TOX4, and β-actin. **E.** Cells were analyzed as in panel D. Corresponding densitometric measurement of phospho DNA-PK Ser-2056 corrected by β-actin was shown. Statistical significance was analyzed using an unpaired two-tailed Student's t-test (**p<0.01). **F.** HeLa cells were treated with TOX4 siRNA and cisplatin (10 μM) for 24 hr, as indicated. The cell lysates were analyzed by immunoblotting for DNA-PKcs phospho-Ser-2056, DNA-PKcs, TOX4, γ-H2AX, and β-actin. **G.** Cells were analyzed as in panel F. Corresponding densitometric measurement of phospho DNA-PK Ser-2056 corrected by β-actin was shown. Statistical significance was analyzed using an unpaired two-tailed Student's t-test (***p<0.001). **H.** HeLa cells were transfected with TOX4-ΔC segment, and treated with cisplatin, as indicated. The cell lysates were analyzed by immunoblotting for DNA-PKcs phospho-Ser-2056, DNA-PKcs, GFP and β-actin.

**Figure S4. The functional association of TOX4 and PNUTS. A.** HeLa cells were treated without or with TOX4 siRNA, PNUTS siRNA for 24 hr, as indicated. Cells were then exposed to (DOX, 5 μM) for 4 hr, and harvested for analysis. Immunoblots of γ-H2AX, TOX4, PNUTS, and β-actin are shown. Corresponding densitometric quantification is shown in the lower panel. **B.** TOX4 was not required for the induction of PARylation after DNA damage. HeLa cells were treated with TOX4 siRNA and $H_2O_2$ (1 μM) for 5min, 10min and 20min. The cell lysates were analyzed by immunoblotting for PAR and $\alpha$-tubulin.

**Figure S5. TOX4 promotes treatment resistance in cancer. A.** Kaplan-Meier survival analysis of stomach cancer was performed, in groups with high or low expression of TOX4. **B.** Kaplan-Meier survival analysis of urothelial cancer was performed, in groups with high or low expression of TOX4. **C.** Kaplan-Meier survival analysis of pancreatic cancer was performed, in groups with high or low expression of TOX4.

**Figure S6. TOX1 and TOX4 depletion showed the opposite effect on DNA-PKcs activation. A.** HeLa cells were treated with TOX4 siRNA, TOX1 siRNA, and doxorubicin (DOX, 5 μM) for 4 hr, as indicated. The cell lysates were analyzed by immunoblotting for phospho-DNA-PKcs Ser-2056, caspase-3, and β-actin. **B.** Cells were analyzed as in panel A. Corresponding densitometric measurement of phospho-DNA-PKcs Ser-2056 corrected by β-actin was shown. Statistical significance was analyzed using an unpaired two-tailed Student's t-test (**p<0.01).

**Movie S1. GFP-TOX4 is recruited to sites of DNA damage.** HeLa cells expressing GFP-TOX4 were subjected to laser micro-irradiation (system 1). Live cell imaging was performed for 10 min at 10 sec intervals.

**Table S1. TOX4 associates with KU proteins.** GFP-TOX4 was expressed in HeLa cells. GFP IP was performed in cell lysates, and the product subjected to proteomic identification of the associated proteins. A control IP was performed without GFP antibody, and non-specific binding proteins identified in the control IP were removed from the list.

# Figure S1

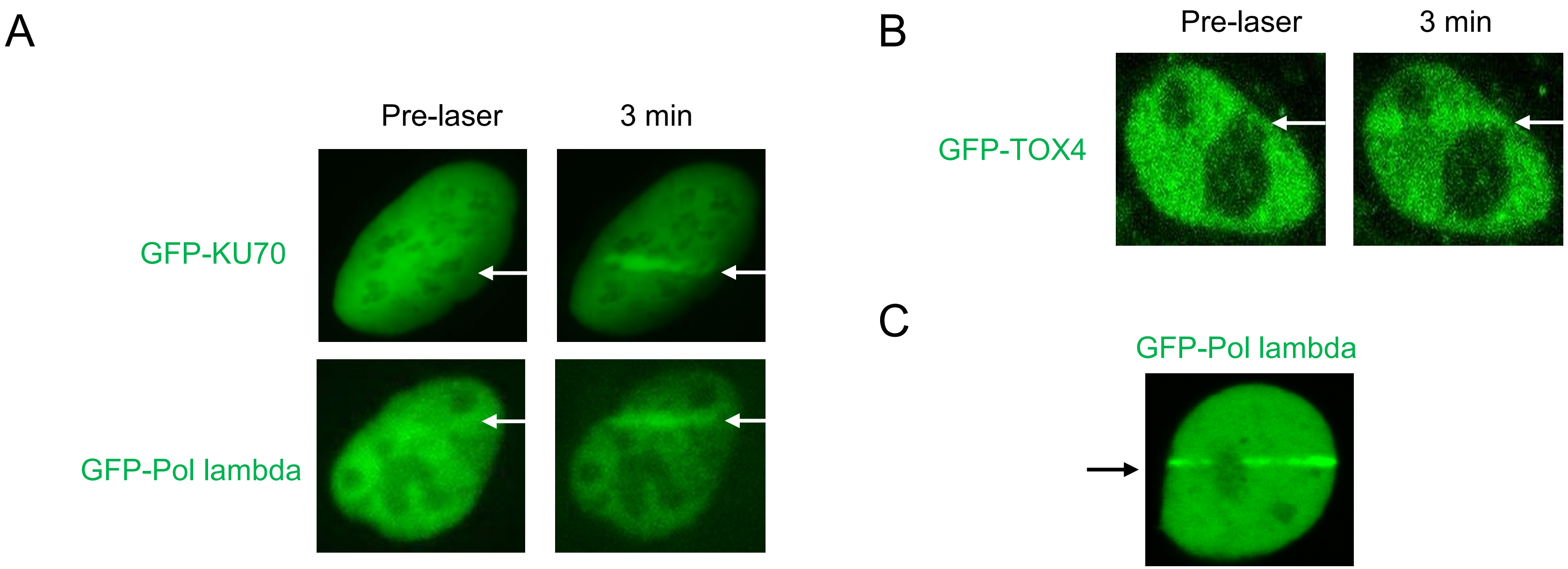
A
Pre-laser
3 min
GFP-KU70
GFP-Pol lambda
B
Pre-laser
3 min
GFP-TOX4
C
GFP-Pol lambda

# Figure S2

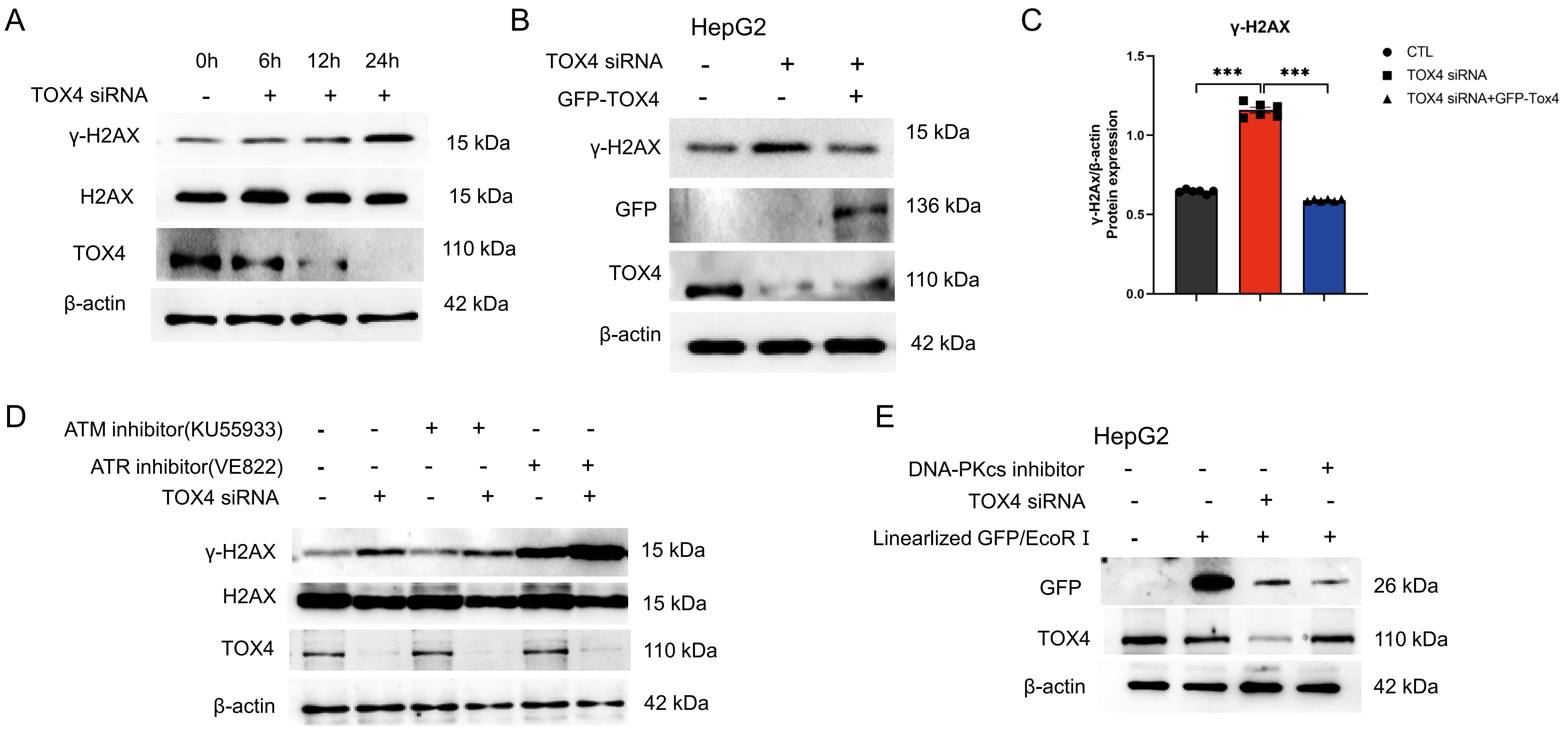
A
0h
6h
12h
24h
TOX4 siRNA
-
+
+
+
γ-H2AX
H2AX
TOX4
β-actin
15 kDa
15 kDa
110 kDa
42 kDa
B
HepG2
TOX4 siRNA
GFP-TOX4
γ-H2AX
GFP
TOX4
β-actin
15 kDa
136 kDa
110 kDa
42 kDa
C
γ-H2AX
γ-H2Ax/β-actin
Protein expression
1.5
1.0
0.5
0.0
***
***
CTL
TOX4 siRNA
TOX4 siRNA+GFP-Tox4
D
ATM inhibitor(KU55933)
ATR inhibitor(VE822)
TOX4 siRNA
γ-H2AX
H2AX
TOX4
β-actin
15 kDa
15 kDa
110 kDa
42 kDa
E
HepG2
DNA-PKcs inhibitor
TOX4 siRNA
Linearlized GFP/EcoR I
GFP
TOX4
β-actin
26 kDa
110 kDa
42 kDa

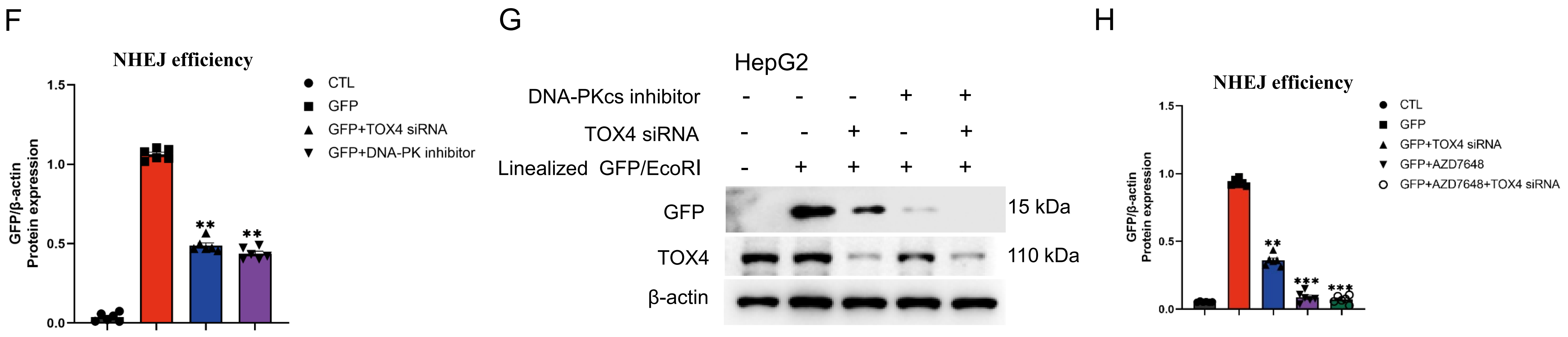
F
NHEJ efficiency
GFP/β-actin
Protein expression
1.5
1.0
0.5
0.0
**
**
CTL
GFP
GFP+TOX4 siRNA
GFP+DNA-PK inhibitor
G
HepG2
DNA-PKcs inhibitor - - - + +
TOX4 siRNA - - + - +
Linealized GFP/EcoRI - + + + +
GFP
TOX4
β-actin
15 kDa
110 kDa
H
NHEJ efficiency
GFP/β-actin
Protein expression
1.5
1.0
0.5
0.0
**
***
***
CTL
GFP
GFP+TOX4 siRNA
GFP+AZD7648
GFP+AZD7648+TOX4 siRNA

# Figure S3

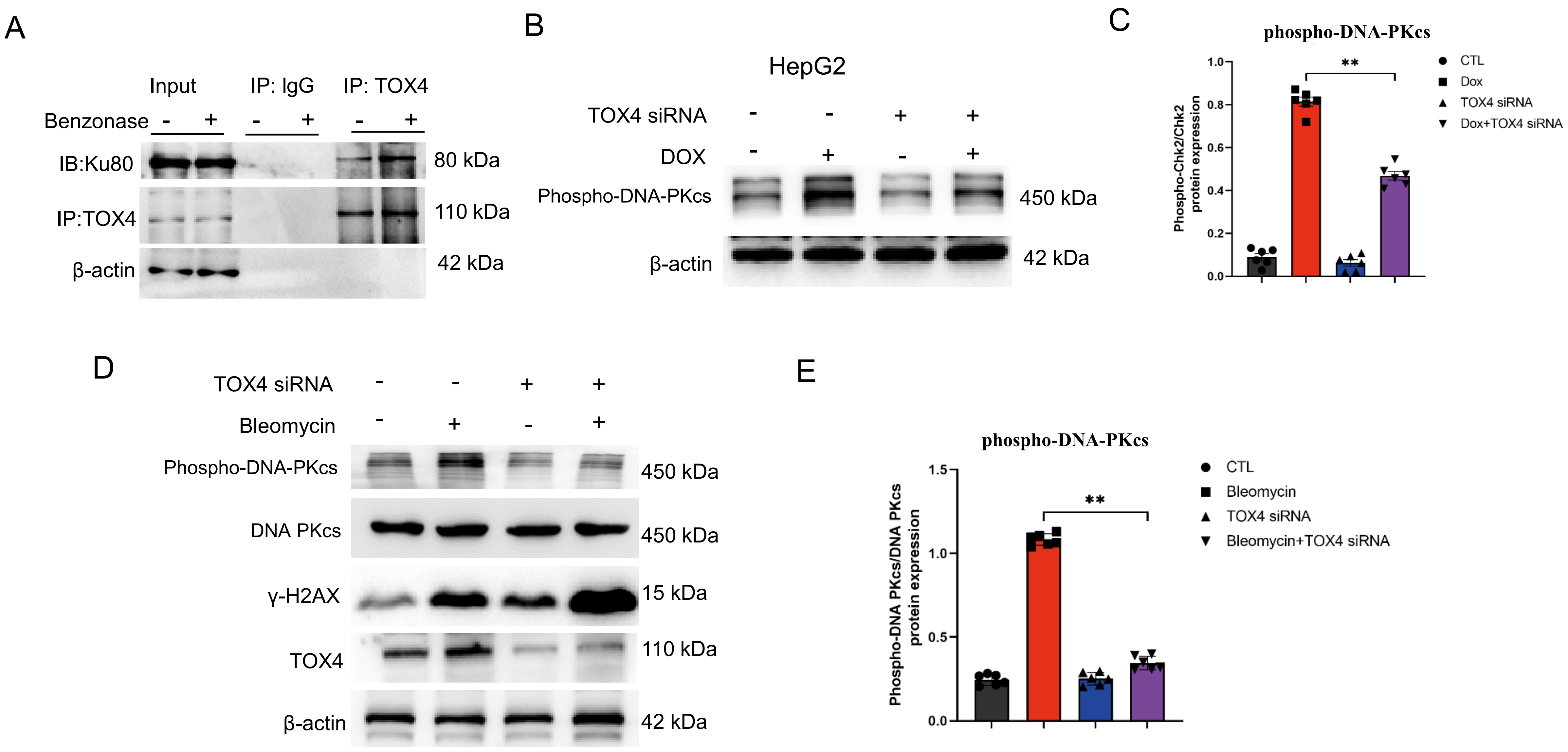
A
Input
IP: IgG
IP: TOX4
Benzonase - + - + - +
IB:Ku80
IP:TOX4
β-actin
80 kDa
110 kDa
42 kDa
B
HepG2
TOX4 siRNA - - + +
DOX - + - +
Phospho-DNA-PKcs
β-actin
450 kDa
42 kDa
C
phospho-DNA-PKcs
Phospho-Chk2/Chk2 protein expression
1.0
0.8
0.6
0.4
0.2
0.0
**
CTL
Dox
TOX4 siRNA
Dox+TOX4 siRNA
D
TOX4 siRNA - - + +
Bleomycin - + - +
Phospho-DNA-PKcs
DNA PKcs
γ-H2AX
TOX4
β-actin
450 kDa
450 kDa
15 kDa
110 kDa
42 kDa
E
phospho-DNA-PKcs
Phospho-DNA PKcs/DNA PKcs protein expression
1.5
1.0
0.5
0.0
**
CTL
Bleomycin
TOX4 siRNA
Bleomycin+TOX4 siRNA

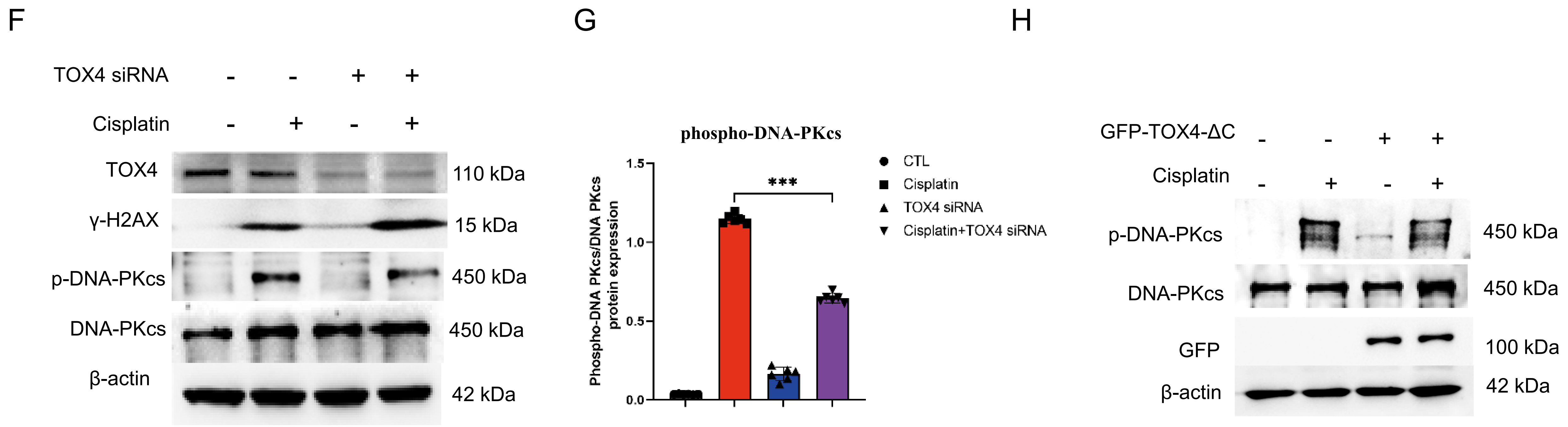
F
TOX4 siRNA - - + +
Cisplatin - + - +
TOX4
110 kDa
γ-H2AX
15 kDa
p-DNA-PKcs
450 kDa
DNA-PKcs
450 kDa
β-actin
42 kDa
G
phospho-DNA-PKcs
Phospho-DNA PKcs/DNA PKcs protein expression
1.5
1.0
0.5
0.0
***
CTL
Cisplatin
TOX4 siRNA
Cisplatin+TOX4 siRNA
H
GFP-TOX4-ΔC - - + +
Cisplatin - + - +
p-DNA-PKcs
450 kDa
DNA-PKcs
450 kDa
GFP
100 kDa
β-actin
42 kDa

Figure S4

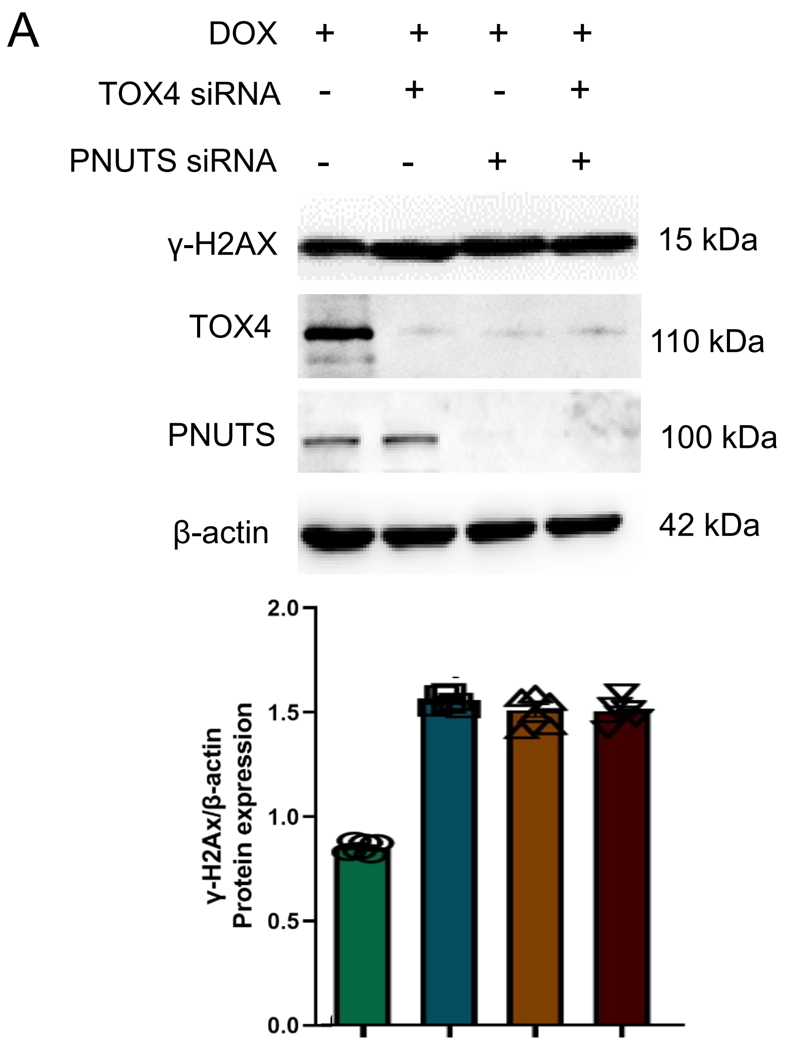
A
DOX + + + +
TOX4 siRNA - + - +
PNUTS siRNA - - + +
γ-H2AX
15 kDa
TOX4
110 kDa
PNUTS
100 kDa
β-actin
42 kDa
γ-H2Ax/β-actin
Protein expression
2.0
1.5
1.0
0.5
0.0

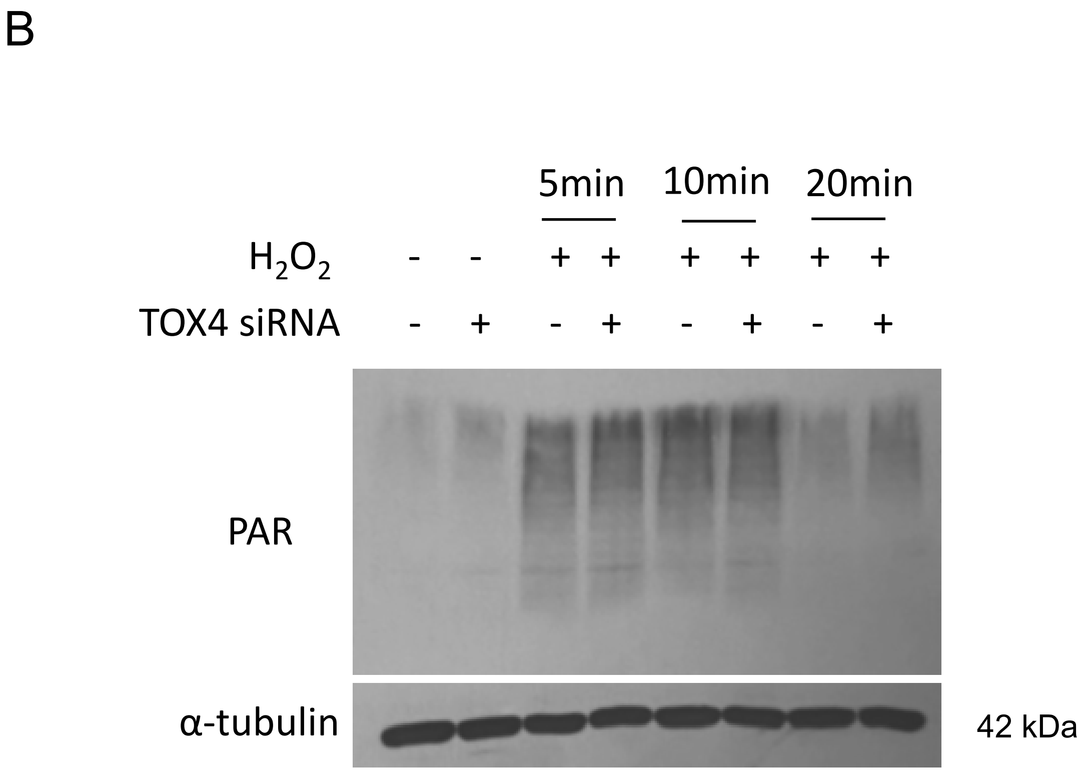
B
5min
10min
20min
H2O2 - - + + + + + +
TOX4 siRNA - + - + - + - +
PAR
α-tubulin
42 kDa

Figure S5

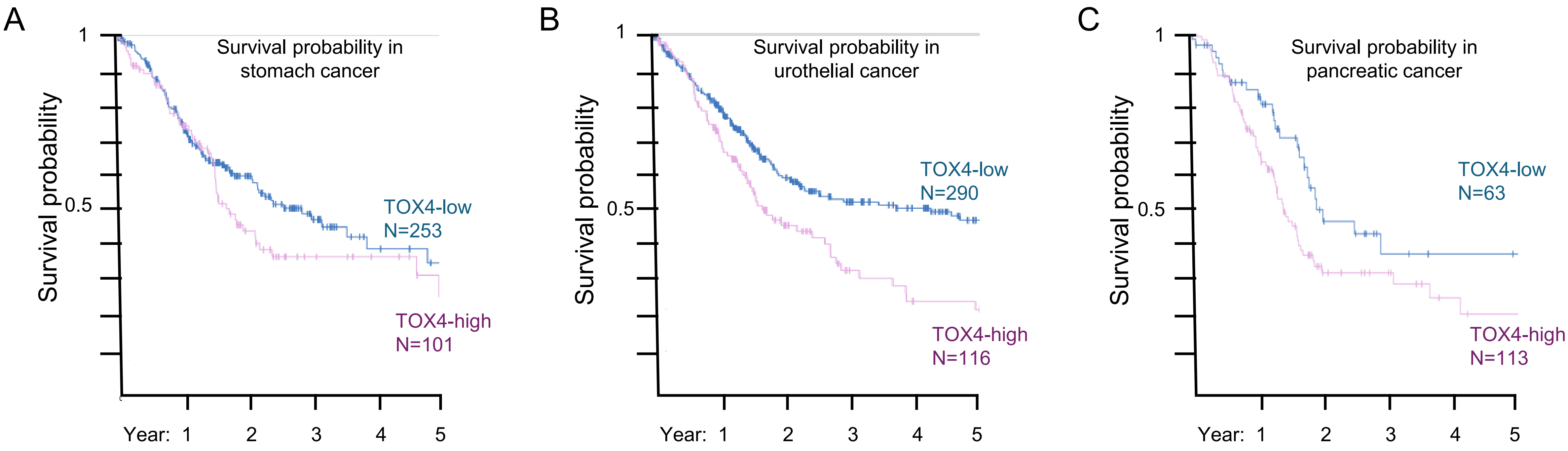
A
1
0.5
Survival probability
Survival probability in
stomach cancer
TOX4-low
N=253
TOX4-high
N=101
Year: 1
2
3
4
5
B
1
0.5
Survival probability
Survival probability in
urothelial cancer
TOX4-low
N=290
TOX4-high
N=116
Year: 1
2
3
4
5
C
1
0.5
Survival probability
Survival probability in
pancreatic cancer
TOX4-low
N=63
TOX4-high
N=113
Year: 1
2
3
4
5

Figure S6

A

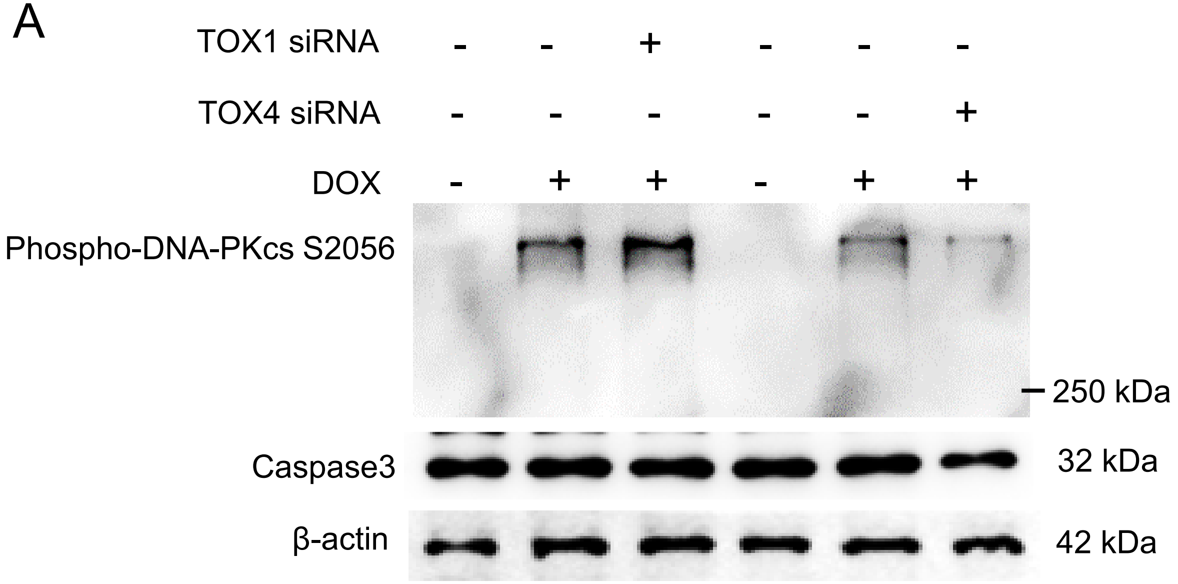

TOX1 siRNA - - + - - -
TOX4 siRNA - - - - - +
DOX - + + - + +
Phospho-DNA-PKcs S2056
250 kDa
Caspase3
32 kDa
β-actin
42 kDa


B

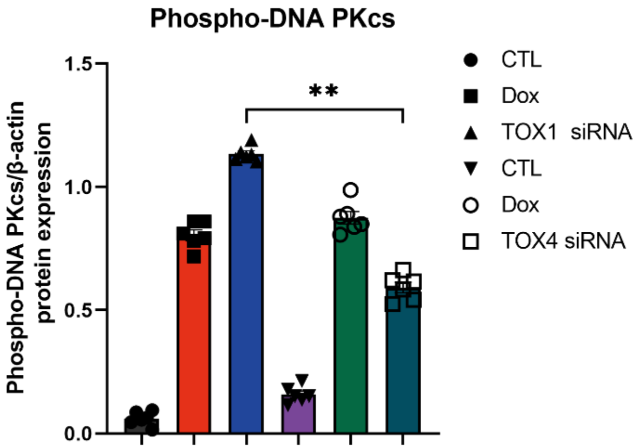

Phospho-DNA PKcs
Phospho-DNA PKcs/β-actin protein expression
1.5
1.0
0.5
0.0
**
CTL
Dox
TOX1 siRNA
CTL
Dox
TOX4 siRNA
