# Supplementary material for: TOX High-Mobility Group Box Family Member 4 promotes DNA double-strand break repair via nonhomologous end joining
Source: J Biol Chem. 2025 May 4;301(6):110174. doi: 10.1016/j.jbc.2025.110174 (PMC12166427; doi:10.1016/j.jbc.2025.110174)
Supplement: Tox4 mass spec [file mmc2.pdf]

true	Empty	Alpha-2-macroglobulin OS=Homo sapiens OX=9606 GN=A2M PE=1 SV=3	A2MG_HUMAN	A2M	163 kDa	100% (8)
true	Empty	Microtubule-associated protein 1A OS=Homo sapiens OX=9606 GN=MAP1A PE=1 SV=6	MAP1A_HUMAN	MAP1A	305 kDa	100% (6)
true	Empty	Coiled-coil domain-containing protein 124 OS=Homo sapiens OX=9606 GN=CCDC124 PE=1 SV=1	CC124_HUMAN	CCDC124	26 kDa	99% (5)
true	Empty	Polymerase delta-interacting protein 3 OS=Homo sapiens OX=9606 GN=POLDIP3 PE=1 SV=2	PDIP3_HUMAN	POLDIP3	46 kDa	100% (5)
true	Empty	TOX high mobility group box family member 4 OS=Homo sapiens OX=9606 GN=TOX4 PE=1 SV=1	TOX4_HUMAN	TOX4	66 kDa	100% (4)
true	Empty	RNA-binding protein 39 OS=Homo sapiens OX=9606 GN=RBM39 PE=1 SV=2	RBM39_HUMAN	RBM39	59 kDa	100% (3)
true	Empty	Ensconsin OS=Homo sapiens OX=9606 GN=MAP7 PE=1 SV=1	MAP7_HUMAN	MAP7	84 kDa	100% (3)
true	Empty	Cleavage and polyadenylation specificity factor subunit 1 OS=Homo sapiens OX=9606 GN=CPSF1 PE=1 SV=2	CPSF1_HUMAN	CPSF1	161 kDa	100% (4)
true	Empty	Probable ATP-dependent RNA helicase DDX41 OS=Homo sapiens OX=9606 GN=DDX41 PE=1 SV=2	DDX41_HUMAN	DDX41	70 kDa	100% (3)
true	Empty	40S ribosomal protein S27-like OS=Homo sapiens OX=9606 GN=RPS27L PE=1 SV=3	RS27L_HUMAN (+1)	RPS27L	9 kDa	100% (4)
true	Empty	X-ray repair cross-complementing protein 6 OS=Homo sapiens OX=9606 GN=XRCC6 PE=1 SV=2	XRCC6_HUMAN	XRCC6	70 kDa	100% (3)
true	Empty	Transferrin receptor protein 1 OS=Homo sapiens OX=9606 GN=TFRC PE=1 SV=2	TFR1_HUMAN	TFRC	85 kDa	100% (2)
true	Empty	Histone H1.2 OS=Homo sapiens OX=9606 GN=HIST1H1C PE=1 SV=2	H12_HUMAN	HIST1H1C	21 kDa	100% (1)
true	Empty	Non-POU domain-containing octamer-binding protein OS=Homo sapiens OX=9606 GN=NONO PE=1 SV=4	NONO_HUMAN	NONO	54 kDa	100% (1)
true	Empty	ATP synthase subunit alpha, mitochondrial OS=Homo sapiens OX=9606 GN=ATP5F1A PE=1 SV=1	ATPA_HUMAN	ATP5F1A	60 kDa	100% (3)
true	Empty	40S ribosomal protein SA OS=Homo sapiens OX=9606 GN=RPSA PE=1 SV=4	RSSA_HUMAN	RPSA	33 kDa	100% (2)
true	Empty	Keratin, type I cytoskeletal 18 OS=Homo sapiens OX=9606 GN=KRT18 PE=1 SV=2	K1C18_HUMAN	KRT18	48 kDa	100% (7)
true	Empty	40S ribosomal protein S3 OS=Homo sapiens OX=9606 GN=RPS3 PE=1 SV=2	RS3_HUMAN	RPS3	27 kDa	100% (2)
true	Empty	40S ribosomal protein S30 OS=Homo sapiens OX=9606 GN=FAU PE=1 SV=1	RS30_HUMAN	FAU	7 kDa	100% (1)
true	Empty	40S ribosomal protein S2 OS=Homo sapiens OX=9606 GN=RPS2 PE=1 SV=2	RS2_HUMAN	RPS2	31 kDa	100% (2)
true	Empty	Myosin light chain 6B OS=Homo sapiens OX=9606 GN=MYL6B PE=1 SV=1	MYL6B_HUMAN (+1)	MYL6B	23 kDa	99% (1)
true	Empty	Peptidyl-prolyl cis-trans isomerase B OS=Homo sapiens OX=9606 GN=PPIB PE=1 SV=2	PPIB_HUMAN	PPIB	24 kDa	100% (2)
true	Empty	Serine/arginine-rich splicing factor 11 OS=Homo sapiens OX=9606 GN=SRSF11 PE=1 SV=1	SRS11_HUMAN	SRSF11	54 kDa	100% (2)
true	Empty	Tripartite motif-containing protein 2 OS=Homo sapiens OX=9606 GN=TRIM2 PE=1 SV=1	TRIM2_HUMAN	TRIM2	82 kDa	100% (2)
true	Empty	60S ribosomal protein L22 OS=Homo sapiens OX=9606 GN=RPL22 PE=1 SV=2	RL22_HUMAN	RPL22	15 kDa	100% (1)
true	Empty	Zinc finger protein 706 OS=Homo sapiens OX=9606 GN=ZNF706 PE=1 SV=1	ZN706_HUMAN	ZNF706	8 kDa	100% (2)
true	Empty	60S ribosomal protein L11 OS=Homo sapiens OX=9606 GN=RPL11 PE=1 SV=2	RL11_HUMAN	RPL11	20 kDa	100% (1)
true	Empty	Creatine kinase S-type, mitochondrial OS=Homo sapiens OX=9606 GN=CKMT2 PE=1 SV=2	KCRS_HUMAN	CKMT2	48 kDa	100% (1)
true	Empty	Tripartite motif-containing protein 3 OS=Homo sapiens OX=9606 GN=TRIM3 PE=1 SV=2	TRIM3_HUMAN	TRIM3	81 kDa	100% (1)
true	Empty	ATP-dependent RNA helicase DHX8 OS=Homo sapiens OX=9606 GN=DHX8 PE=1 SV=1	DHX8_HUMAN	DHX8	139 kDa	100% (2)
true	Empty	14-3-3 protein zeta/delta OS=Homo sapiens OX=9606 GN=YWHAZ PE=1 SV=1	1433Z_HUMAN	YWHAZ	28 kDa	100% (1)
true	Empty	Guanine nucleotide-binding protein-like 3-like protein OS=Homo sapiens OX=9606 GN=GNL3L PE=1 SV=1	GNL3L_HUMAN	GNL3L	66 kDa	100% (1)
true	Empty	pre-mRNA 3' end processing protein WDR33 OS=Homo sapiens OX=9606 GN=WDR33 PE=1 SV=2	WDR33_HUMAN	WDR33	146 kDa	99% (2)
true	Empty	U4/U6.U5 tri-snRNP-associated protein 1 OS=Homo sapiens OX=9606 GN=SART1 PE=1 SV=1	SNUT1_HUMAN	SART1	90 kDa	99% (1)
true	Empty	ATP synthase F(0) complex subunit C1, mitochondrial OS=Homo sapiens OX=9606 GN=ATP5MC1 PE=1 SV=2	AT5G1_HUMAN (+2)	ATP5MC1	14 kDa	99% (2)
true	Empty	Chromobox protein homolog 8 OS=Homo sapiens OX=9606 GN=CBX8 PE=1 SV=3	CBX8_HUMAN	CBX8	43 kDa	99% (2)
true	Empty	Protein FAM32A OS=Homo sapiens OX=9606 GN=FAM32A PE=1 SV=2	FA32A_HUMAN	FAM32A	13 kDa	99% (2)
true	Empty	60S ribosomal protein L31 OS=Homo sapiens OX=9606 GN=RPL31 PE=1 SV=1	RL31_HUMAN	RPL31	14 kDa	99% (2)
true	Empty	60S ribosomal protein L35 OS=Homo sapiens OX=9606 GN=RPL35 PE=1 SV=2	RL35_HUMAN	RPL35	15 kDa	99% (2)
true	Empty	Small nuclear ribonucleoprotein Sm D3 OS=Homo sapiens OX=9606 GN=SNRPD3 PE=1 SV=1	SMD3_HUMAN	SNRPD3	14 kDa	99% (2)
true	Empty	Survival of motor neuron-related-splicing factor 30 OS=Homo sapiens OX=9606 GN=SMNDC1 PE=1 SV=1	SPF30_HUMAN	SMNDC1	27 kDa	99% (2)
true	Empty	Zinc finger C2HC domain-containing protein 1A OS=Homo sapiens OX=9606 GN=ZC2HC1A PE=1 SV=2	ZC21A_HUMAN	ZC2HC1A	35 kDa	99% (1)
true	Empty	40S ribosomal protein S4, X isoform OS=Homo sapiens OX=9606 GN=RPS4X PE=1 SV=2	RS4X_HUMAN	RPS4X	30 kDa	99% (2)
true	Empty	X-ray repair cross-complementing protein 5 OS=Homo sapiens OX=9606 GN=XRCC5 PE=1 SV=3	XRCC5_HUMAN	XRCC5	83 kDa	99% (1)
true	Empty	Cleavage and polyadenylation specificity factor subunit 7 OS=Homo sapiens OX=9606 GN=CPSF7 PE=1 SV=1	CPSF7_HUMAN	CPSF7	52 kDa	99% (1)
true	Empty	Disco-interacting protein 2 homolog B OS=Homo sapiens OX=9606 GN=DIP2B PE=1 SV=3	DIP2B_HUMAN	DIP2B	171 kDa	99% (1)
true	Empty	Cytoplasmic dynein 1 heavy chain 1 OS=Homo sapiens OX=9606 GN=DYNC1H1 PE=1 SV=5	DYHC1_HUMAN	DYNC1H1	532 kDa	99% (1)
true	Empty	Trifunctional enzyme subunit alpha, mitochondrial OS=Homo sapiens OX=9606 GN=HADHA PE=1 SV=2	ECHA_HUMAN	HADHA	83 kDa	99% (1)
true	Empty	Trifunctional enzyme subunit beta, mitochondrial OS=Homo sapiens OX=9606 GN=HADHB PE=1 SV=3	ECHB_HUMAN	HADHB	51 kDa	99% (1)
true	Empty	Elongation factor Tu, mitochondrial OS=Homo sapiens OX=9606 GN=TUFM PE=1 SV=2	EFTU_HUMAN	TUFM	50 kDa	99% (1)
true	Empty	Histone H2A type 1-A OS=Homo sapiens OX=9606 GN=HIST1H2AA PE=1 SV=3	H2A1A_HUMAN (+14)	HIST1H2AA	14 kDa	99% (1)
true	Empty	Heterogeneous nuclear ribonucleoprotein H3 OS=Homo sapiens OX=9606 GN=HNRNPH3 PE=1 SV=2	HNRH3_HUMAN	HNRNPH3	37 kDa	99% (1)
true	Empty	Heat shock protein HSP 90-alpha A2 OS=Homo sapiens OX=9606 GN=HSP90AA2P PE=1 SV=2	HS902_HUMAN	HSP90AA2P	39 kDa	99% (1)
true	Empty	Integrin beta-1 OS=Homo sapiens OX=9606 GN=ITGB1 PE=1 SV=2	ITB1_HUMAN	ITGB1	88 kDa	99% (1)
true	Empty	cAMP-dependent protein kinase catalytic subunit alpha OS=Homo sapiens OX=9606 GN=PRKACA PE=1 SV=2	KAPCA_HUMAN (+1)	PRKACA	41 kDa	99% (1)
true	Empty	Creatine kinase B-type OS=Homo sapiens OX=9606 GN=CKB PE=1 SV=1	KCRB_HUMAN	CKB	43 kDa	99% (1)
true	Empty	Myoglobin OS=Homo sapiens OX=9606 GN=MB PE=1 SV=2	MYG_HUMAN	MB	17 kDa	99% (1)
true	Empty	Nucleosome assembly protein 1-like 4 OS=Homo sapiens OX=9606 GN=NAP1L4 PE=1 SV=1	NP1L4_HUMAN	NAP1L4	43 kDa	99% (1)

true	Empty	Phosphoglycerate kinase 2 OS=Homo sapiens OX=9606 GN=PGK2 PE=1 SV=3	PGK2_HUMAN	PGK2	45 kDa	99% (1)
true	Empty	Ras-related protein Rap-1A OS=Homo sapiens OX=9606 GN=RAP1A PE=1 SV=1	RAP1A_HUMAN	RAP1A	21 kDa	99% (1)
true	Empty	60S ribosomal protein L17 OS=Homo sapiens OX=9606 GN=RPL17 PE=1 SV=3	RL17_HUMAN	RPL17	21 kDa	99% (1)
true	Empty	60S ribosomal protein L8 OS=Homo sapiens OX=9606 GN=RPL8 PE=1 SV=2	RL8_HUMAN	RPL8	28 kDa	99% (1)
true	Empty	40S ribosomal protein S13 OS=Homo sapiens OX=9606 GN=RPS13 PE=1 SV=2	RS13_HUMAN	RPS13	17 kDa	99% (1)
true	Empty	Superoxide dismutase [Mn], mitochondrial OS=Homo sapiens OX=9606 GN=SOD2 PE=1 SV=3	SODM_HUMAN	SOD2	25 kDa	99% (1)
true	Empty	Splicing factor 45 OS=Homo sapiens OX=9606 GN=RBM17 PE=1 SV=1	SPF45_HUMAN	RBM17	45 kDa	99% (1)
true	Empty	T-complex protein 1 subunit gamma OS=Homo sapiens OX=9606 GN=CCT3 PE=1 SV=4	TCPG_HUMAN	CCT3	61 kDa	99% (1)
true	Empty	THO complex subunit 1 OS=Homo sapiens OX=9606 GN=THOC1 PE=1 SV=1	THOC1_HUMAN	THOC1	76 kDa	99% (1)
true	Empty	Troponin C, slow skeletal and cardiac muscles OS=Homo sapiens OX=9606 GN=TNNC1 PE=1 SV=1	TNNC1_HUMAN	TNNC1	18 kDa	99% (1)
true	Empty	Tubulin polymerization-promoting protein OS=Homo sapiens OX=9606 GN=TPPP PE=1 SV=1	TPPP_HUMAN	TPPP	24 kDa	99% (1)
true	Empty	Hemoglobin subunit gamma-1 OS=Homo sapiens OX=9606 GN=HBG1 PE=1 SV=2	HBG1_HUMAN	HBG1	16 kDa	99% (1)
